# Chromosome-level genome provides insights into environmental adaptability and innate immunity in the common dolphin *(Delphinus delphis)*

**DOI:** 10.1186/s12864-024-10268-4

**Published:** 2024-04-16

**Authors:** Kui Ding, Qinzeng Xu, Liyuan Zhao, Yixuan Li, Zhong Li, Wenge Shi, Qianhui Zeng, Xianyan Wang, Xuelei Zhang

**Affiliations:** 1 Key Laboratory of Marine Eco-Environmental Science and Technology, First Institute of Oceanography, Ministry of Natural Resources, Qingdao, China; 2 Laboratory for Marine Ecology and Environmental Science, Qingdao Marine Science and Technology Center, Qingdao, China; 3 Key Laboratory of Marine Ecological Conservation and Restoration, Third Institute of Oceanography, Ministry of Natural Resources, Xiamen, China; 4 National Engineering Laboratory for Integrated Aero-Space-Ground-Ocean Big Data Application Technology, Xi’an, China

**Keywords:** *Delphinus delphis*, Genome sequencing, Chromosome assembly, Comparative genomics, Demographic history

## Abstract

The common dolphin (*Delphinus delphis*) is widely distributed worldwide and well adapted to various habitats. Animal genomes store clues about their pasts, and can reveal the genes underlying their evolutionary success. Here, we report the first high-quality chromosome-level genome of *D. delphis*. The assembled genome size was 2.56 Gb with a contig N50 of 63.85 Mb. Phylogenetically, *D. delphis* was close to *Tursiops truncatus* and *T. aduncus*. The genome of *D. delphis* exhibited 428 expanded and 1,885 contracted gene families, and 120 genes were identified as positively selected. The expansion of the HSP70 gene family suggested that *D. delphis* has a powerful system for buffering stress, which might be associated with its broad adaptability, longevity, and detoxification capacity. The expanded IFN-α and IFN-ω gene families, as well as the positively selected genes encoding tripartite motif-containing protein 25, peptidyl-prolyl cis-trans isomerase NIMA-interacting 1, and p38 MAP kinase, were all involved in pathways for antiviral, anti-inflammatory, and antineoplastic mechanisms. The genome data also revealed dramatic fluctuations in the effective population size during the Pleistocene. Overall, the high-quality genome assembly and annotation represent significant molecular resources for ecological and evolutionary studies of *Delphinus* and help support their sustainable treatment and conservation.

**Supplementary Information:**

The online version contains supplementary material available at 10.1186/s12864-024-10268-4.

## Introduction

The common dolphin (*Delphinus delphis*), recognized as one of the most widely distributed small cetaceans, inhabits seas ranging from temperate to tropical regions globally [1, 2]. Characterized by pelagic habitats [3, 4], these dolphins exhibit remarkable diving abilities, reaching depths up to 200 m [4], and demonstrate exceptional mobility, capable of covering over 500 km within days [5–7]. Notably, their strong adaptability is reflected in their diverse diet and resilience to environmental changes, as evidenced by recent studies on genotype-environment associations [8–10]. Despite their position at the top of the marine food chain, which subjects them to biomagnification of pollutants, common dolphins show no significant effects from contaminant accumulation [11–13]. Common dolphins, characterized as K-strategists—species that allocate substantial resources towards nurturing a smaller number of offspring over extended lifespans—are believed to have a lifespan ranging from 25 to 30 years [6, 14]. Research utilizing logistic regression methods has determined the onset of sexual maturity and initial reproductive age to be approximately 8.24 and 9.23 years, respectively [15]. Moreover, the duration of their reproductive phase is estimated to span 10 to 20 years [11, 16, 17]. Their resilience, coupled with their K-strategist reproductive strategy, underscores the need for further research into their immune system and environmental adaptability.

The innate immune system, often heralded as the first barrier against pathogenic invasion, relies on a swift, non-specific mechanism encompassing cytokine interactions, pattern recognition receptors, the complement system, and a variety of immune cells tasked with pathogen identification and elimination [18, 19]. Despite considerable research efforts into the immunology of marine mammals over the past decades, our understanding of cetacean immune systems remains notably fragmented [20]. The lymphoid organs in cetaceans, subject to the impacts of infectious agents and inflammatory diseases, have seen epidemics and isolated instances of distemper-like illnesses across species, including common dolphins [20, 21]. The advent of comprehensive genome analysis has shed light on the significance of certain genes and pathways within cetacean immune responses, enriching our grasp of their immune defenses [22]. While instances of primary hematopoietic neoplasms and lymphoproliferative diseases are relatively rare in cetaceans [23], sporadic reports of malignant lymphoma have emerged in a limited number of dolphin species [24, 25]. In beluga whales, an uptick in neoplastic diseases linked to exposure to persistent organic pollutants hints at potentially compromised antitumoral immune responses or carcinogenic effects stemming from environmental contaminants [26, 27]. Nevertheless, it’s important to highlight that instances of neoplastic disease have yet to be identified in common dolphins.

Reference genomes serve as pivotal tools for delving into the evolutionary relationships, historical demographics, and the evolutionary journey of genes and traits within the animal kingdom [28]. Within the marine ecosystem, cetaceans hold a distinctive role, underscored by the assembly and publication of multiple reference genomes in recent years (e.g., *Delphinapterus leucas*, *Orcinus orca*, *T. truncatus*, *Physeter macrocephalus*, etc.), which have significantly advanced our grasp of their evolutionary narratives [29, 30]. These initiatives have paved the way for comparative genomic and evolutionary studies, broadening our comprehension of these exceptional marine inhabitants. Despite these advancements, there remains a gap in the availability of high-quality, chromosome-level genomes for several marine mammal species, essential for nuanced analyses of ecology and biological evolution [31–33].

This study presents the assembly and annotation of a high-quality, chromosome-level genome for the common dolphin (*D. delphis*), achieved through the synergistic application of Illumina sequencing, PacBio-circular consensus sequencing (CCS), and Hi-C technology. Our comparative genomic analyses offer new insights into the phylogenetic positioning, demographic history, and genetic traits pivotal for adaptation to marine environments and innate immunity. The unveiling of this well-annotated, high-caliber genome assembly not only propels forward comparative genomic research but also lays a solid foundation for biodiversity cataloging and supports informed conservation and management strategies for cetaceans.

## Materials and methods

### Sample collection and DNA and RNA extraction

On January 7, 2019, a male common dolphin (*D. delphis*, specimen ID Code Ddel79) was found stranded along the coast of Lianjiang, Fujian Province, China. This specimen, displaying a fresh odor, intact appearance with some superficial skin abrasions, and clear eyes, was determined to be less than 24 h post-mortem (classified as Code 2) [34]. It was promptly frozen for preservation and identified as a short-beaked common dolphin based on its physical characteristics [35] and mitochondrial DNA markers (cox1 and cytb). We harvested fresh samples from the muscle, skin, heart, adipose tissue, liver, and brain. The muscle tissue was immediately flash-frozen in liquid nitrogen and stored at -80℃ for DNA extraction. The other tissues were submerged in RNA preservation solution (Absin Bioscience Inc.) to maintain RNA integrity. The genomic DNA was extracted using the standard phenol-chloroform method and assessed for quality and integrity through 1% agarose gel electrophoresis, Qubit 4 (Invitrogen, USA), and Agilent qPCR (Mx3005P, USA). Only DNA samples that met high-quality standards were selected for library preparation. Total RNA was extracted from the remaining tissues using the RNeasy Mini Kit (Qiagen, Germany), in accordance with the manufacturer’s instructions. The quality and integrity of the RNA were evaluated using Agilent qPCR. RNA samples with an RNA Integrity Number (RIN) of at least 5.3 and a total amount of at least 0.4 µg were selected for further transcription and library sequencing.

### Library construction and sequencing

Short-insert paired-end (PE) (350 bp) DNA Illumina library was constructed according to manufacturer’s instructions (Illumina, San Diego, California, US). Sequencing runs for the PE library were performed on the Illumina Novaseq 6000 platform. PacBio-CCS sequencing libraries were prepared using the SMRTbell Template Prep Kit 1.0-SPv3 according to standard protocols. The Hi-C library, targeting *D. delphis* muscle tissues, was constructed in alignment with the methodology outlined by Belton et al. (2012) [36], facilitating the achievement of a chromosome-level genome assembly. Furthermore, RNA sequencing was carried out on samples from five primary tissues of *D. delphis* to enhance the genome annotation process.

### Genome assembly

The genome size, heterozygosity, and repeat rate of *D. delphis* were estimated using a K-mer approach (k = 17) [37] based on the Jellyfish method [38]. To refine our estimates, we subsequently applied GenomeScope2 [39] for a more precise analysis. For assembly, we utilized long-read data with Hifiasm (v0.8-dirty-r280) [40] under default settings to generate contig sequences (*p_ctg.fa). These sequences were further processed with Purge Haplotigs (v1.1.0) [41] to reduce heterozygosity by identifying and removing haplotigs based on low-coverage areas in the genome bam file derived from second-generation sequencing data, yielding optimized contig sequences.

To obtain valid Hi-C data, we aligned the contig sequences of the *D. delphis* genome using bowtie2 v2.4.2 [42] and hicup v0.8.1 [43]. Specifically, we selected reads near the enzyme digestion sites to facilitate the assembly process. Subsequently, the contig sequences were clustered, ordered, and oriented with ALLHIC v0.9.8 [40, 44], based on their Hi-C interaction data. Utilizing methodologies delineated in Howe et al., 2021 [45], and employing the Rapid Curation pipeline from the Sanger Institute (https://gitlab.com/wtsi-grit/rapid-curation), in conjunction with the published common dolphin whole-genome reference sequence from NCBI (GCF_949987515.1), the complete X chromosome and a partial Y chromosome of *D. delphis* were successfully assembled. Finally, we manually adjusted the orientation and order of each chromosome using Juicebox to produce a chromosomal-level genome assembly.

To assess the completeness and accuracy of the genome assembly, 248 conserved genes from 6 eukaryotic model organisms were selected and subjected to the Core Eukaryotic Genes Mapping Approach (CEGMA v2.5) [46] analysis and the Benchmarking Universal Single-Copy Orthologs (BUSCO v4.1.2) [47] analysis. We then aligned the assembled genome against high-fidelity (HiFi) reads using Minimap2 [48] to determine the gene collapse rate and identify the extent of base collapse. Coverage depth was calculated using SAMtools v0.1.19 [49], and areas of significantly reduced coverage were pinpointed through a custom Python script. Furthermore, the assembly’s Quality Value (QV) was quantified with Merqury [50], providing an additional measure of assembly integrity.

### Genome annotation

To annotate the repetitive sequences in the *D. delphis* genome, we employed a dual strategy that combines both homology alignment and *de novo* prediction techniques. Tandem repeat elements were pinpointed using Tandem Repeats Finder v4.09 [51], which relies on ab initio prediction for identification. For homology-based alignments, we annotated transposable elements (TEs) using RepeatMasker version 4.0.7, alongside its internal tool RepeatProteinMask [52], by aligning sequences against the RepBase library v14.06 [53]. In parallel, for *de novo* prediction of repeats, tools such as LTR_Finder [54], RepeatModeler version 1.0.10 [55], and RepeatScout v1.0.5 (http://www.repeatmasker.org/) were utilized to construct a comprehensive de novo repeat library. This library was then applied to identify DNA-level repetitive sequences within the *D. delphis* genome, ensuring a thorough and accurate annotation of its repetitive elements.

For predicting protein-coding genes, a comprehensive automated gene prediction pipeline was implemented, utilizing multiple algorithms including Augustus v3.2.3 [56], Geneid v1.4 [46], Genescan v1.0 [57], GlimmerHMM v3.04 [58], and SNAP v2013-11-29 (http://korflab.ucdavis.edu/software.html). The sequences of homologous proteins were downloaded from the NCBI database and DNA Zoo (Table S1). These protein sequences were then aligned to the genome using tblastN v2.2.26 [59] with E-value ≤ 1e-5. Subsequently, the matching proteins were aligned to their corresponding genomic sequences, with GeneWise v2.4.1 [60] facilitating the precise spliced alignments necessary for predicting gene structures within each protein-coding region. Additionally, RNA-seq reads were meticulously aligned to the assembled genome using Hisat v2.0.4 [61] to pinpoint exon regions and splice junctions. The outcomes of these alignments served as crucial input for Cufflinks v2.2.1 [62], which was employed for the assembly of genome-based transcripts.

Gene function annotation was meticulously conducted by aligning the protein sequences to the Swiss-Prot database using BLASTP v2.2.28, with a stringent E-value ≤ 1e-5 [63]. InterProScan v5 [64] was used to annotate the motifs and domains via searches against publicly available databases, including PROSITE [65], Pfam [66], PRINTS [67], PANTHER [68], SMRT [69], and ProDom [70]. This comprehensive approach allowed for the assignment of Gene Ontology (GO) [71] identifiers to genes, correlating them with specific InterPro entries for a nuanced understanding of gene functions. Additionally, protein functions were inferred by leveraging annotation data from the closest matches found in the Swiss-Prot [72] and NR [73] databases, following a BLAST search with an E-value threshold of < 1e-5. The Kyoto Encyclopedia of Genes and Genomes (KEGG) [74] database further augmented gene set annotations, providing a pathway-level perspective of gene functions.

For the comprehensive annotation of non-coding RNAs (ncRNAs), tRNAscan-SE version 1.23 [75] was utilized to identify tRNAs, while other ncRNA types were discerned through alignment against the RFAM database version 12.0 [76], using INFERNAL version 1.1.2 [77]. This dual-faceted approach ensures a thorough annotation of both protein-coding and non-coding elements within the *D. delphis* genome.

### Gene family, phylogenetic analysis, and divergence time estimation

In this study, we selected 7 species from the Odontoceti group, 8 species from the Mysticeti group, and *Ovis aries* as an outgroup for our phylogenetic analysis, ensuring a broad representation across the evolutionary tree previously constructed for these cetacean groups [78, 79]. The complete genome sequences and annotations of *O. aries, P. macrocephalus, T. truncatus, D. leucas, Neophocaena asiaeorientalis, Lipotes vexillifer, Eubalaena glacialis, Balaena mysticetus, Balaenoptera acutorostrata, Balaenoptera musculus, Balaenoptera physalus, Megaptera novaeangliae*, and *Eschrichtius robustus* were acquired from the NCBI and DNA Zoo databases (Table S1). For the genomic data of *Balaenoptera brydei*, *Grampus griseus*, and *T. aduncus*, we relied on datasets made available by their publishers [80]. Prior to incorporating these datasets into our analysis, we meticulously verified the credibility of these sources and performed additional quality assessments to guarantee the integrity of the data. The time calibration points were based on data sourced from the Timetree website (http://www.timetree.org/) and four taxa (*Ovis aries, Odontoceti, Delphinidae*, and *Balaenopteridae*), which were used to calibrate the molecular clock in our phylogenetic analysis (Table S2). For detailed procedures including gene family identification, phylogenetic analysis, and divergence time estimation, along with the specific software used, please refer to the respective sections in the Supplementary Materials and Methods.

### Expansive, contractive, and rapidly evolving gene families

CAFÉ v4.2 [81] with the default parameters was employed to systematically analyze the expansion and contraction within the gene families of *D. delphis*. Changes within gene families were deemed statistically significant for *p*-values less than 0.05, and highly significant for *p*-values less than 0.01, highlighting gene families undergoing notable evolutionary shifts. To elucidate the biological implications of gene family dynamics in *D. delphis*, GO and KEGG [74] were used to perform functional enrichment analyses on both expanded and contracted gene families. Gene families exhibiting rapid evolution, identified through *p*-values < 0.05, were further characterized with functional annotations provided by eggnog-mapper2. These annotations were subsequently analyzed for enrichment patterns using ClusterProfiler v.4 [82].

### Detection of positively selected genes

The identification of positively selected genes (PSGs) in the common dolphin was accomplished through the application of the CodeML v4.9 module within the Phylogenetic Analysis by Maximum Likelihood (PAML) software suite [83]. For other detailed methods and software, please refer to the supplementary materials and methods.

### Homology analysis

To investigate the homology between *D. delphis* (2n = 22) and its sister species, *T. truncatus* (2n = 22), we conducted a chromosomal-level pairwise comparison. This comparison encompassed both coding genes and the entire genome, utilizing JCVI v1.1.22 [84] for coding genes and Mummer v4.0.0rc1 [85] for whole-genome comparison. The detailed methods can be found in the supplementary materials and methods.

### Demographic history reconstruction

The PSMC (pairwise sequentially Markovian coalescent) method v0.6.5-r67 [86] was used to reconstruct the demographic history of *D. delphis*. Firstly, Samtools and bcftools, called with “samtools mpileup -C30” and “vcfutils.pl vcf2fq -d 10 -D 100”, were used to construct diploid genome references. Then the history of changes in the *D. delphis* effective population size were inferred by PSMC with the parameters “-N25 -t15 -r5 -p “4 + 25*2 + 4 + 6"”, as in Warren et al. (2017) [87]. The estimated generation time (g) was set to 9.23 [15] and the mutation rate per generation per site (µ) was 1.5 × 10^− 8^ [78].

## Results

### Genome size estimation, assembly, and annotation

**Fig. 1:**
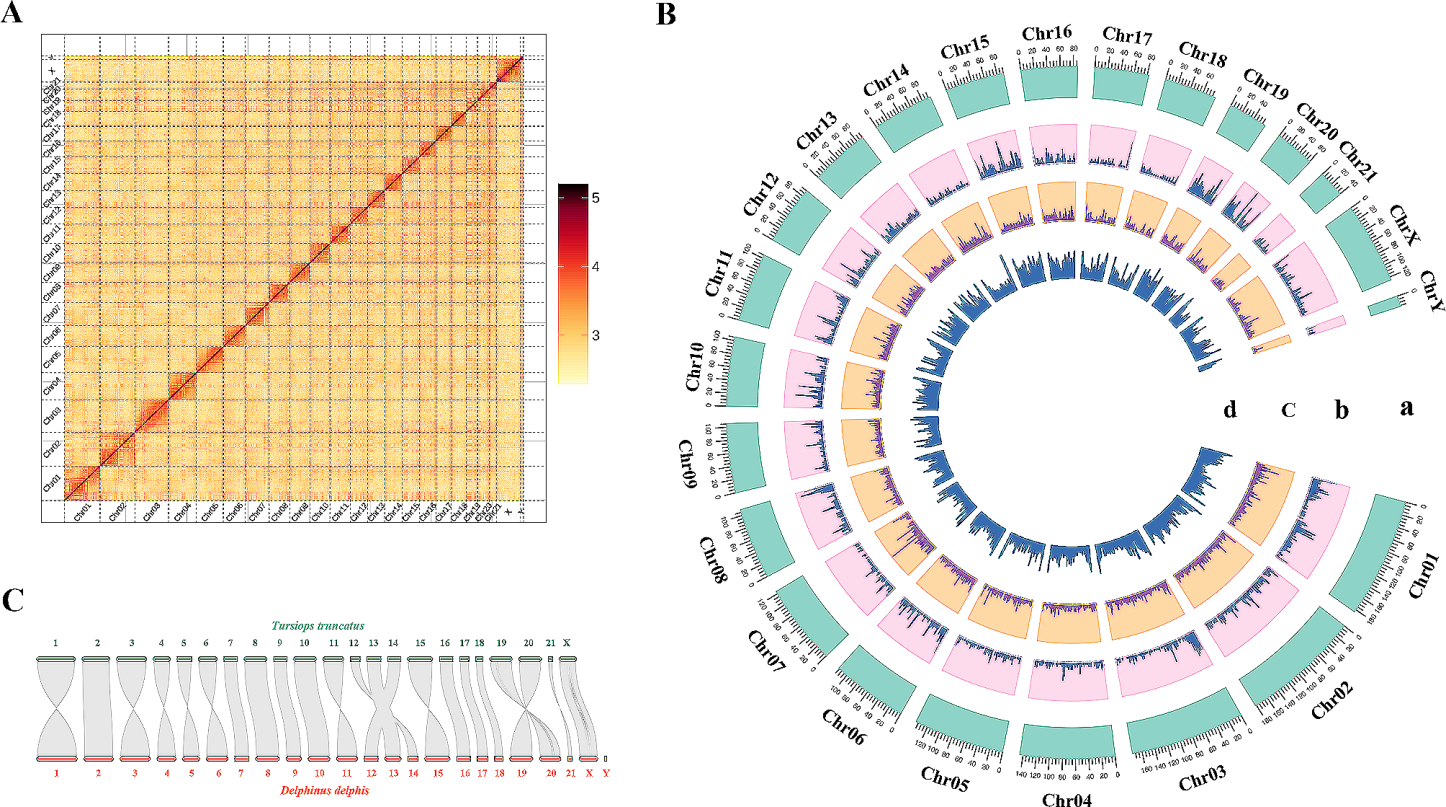
Genome assembly, synteny, and Circos atlas of *D. delphis*. (**A**) Log10-scaled Hi-C interaction heat map of chromatin in *D. delphis* whole genome, indicating interaction intensity by color depth. (**B**) Circos diagram of the genetic information structure of *D. delphis*. From the outer circle to the inner circle: a, chromosome length information; b, gene density mapping results; c, repeat sequence density; d, GC content. (**C**) Genome synteny between *D. delphis* and *T. truncatus*

The genome of a stranded *D. delphis* was sequenced using Illumina and Pacbio technologies. After quality control, we obtained 84.2 Gb Illumina short reads (Table S3). The total number of 17-mers was 75,216,715,516, and the K-mer depth was 29 (Table S4). Therefore, the genome size was estimated at 2569.25 Mb using Jellyfish (Table S4). A subsequent estimation with GenomeScope2 closely matched this at 2,558.37 Mb (Fig. S1). After data processing, a total 80.8 Gb of high-fidelity (HiFi) reads with an average length of 14,404 bp was retained for subsequent analysis (Table S5). The assembly resulted in 266 contigs with a total length of 2,559.1 Mb and a contig N50 of 63.85 Mb. These contigs were organized into 23 chromosomes, including X and a partial Y choromosomes, achieving an anchoring rate of 93.81% (Table 1; Fig. 1A). Finally, the chromosome-level genome assembly covered a total length of 2,400.79 Mb, demonstrating a high level of continuity (Table 1). Moreover, the genome assembly metrics for *D. delphis* were compared with those of *T. truncatus*, as presented in Table 1.

**Table 1:** Comparative Genome Assembly Metrics for *D. delphis* and *T. truncatus*

	Metrics	*D. delphis*	*T. truncatus*
Genome assembly statistics	Contig total length	2,559.1 Mb	2,372.3 Mb
	Number of contigs	266	1036
	Number of scaffolds	187	362
	Maximum contig length	137.3 Mb	58.3 Mb
	Maximum scaffold length	183.5 Mb	183.74 Mb
	N50 length (contigs)	63.9 Mb	9.7 Mb
	N50 length (scaffolds)	108.9 Mb	108.4 Mb
Genome characteristics	Guanine-cytosine (GC) content	41.92%	41.44%
	Predicted heterozygosity	0.33%	0.27%
	Content of repeat sequences	41.78%	34.80%
	Predicted protein-coding gene number	22,148	18,465
	Average exons of predicted protein-coding genes	7.92	9.88
	Predicted non-coding RNA gene number	44,085	12,372
	Quantity of assembled scaffolds anchored on chromosomes	23	22
	Length of scaffolds anchored on chromosomes	2,400.8 Mb	2408.3 Mb
	Chromosome number	23	22
	Total number of predicted genes	22,148	24,128

The assembly accuracy was evaluated using a library of short reads, which showed a high mapping rate of 99.69% against the assembled genome. This was complemented by a coverage rate of 99.96% and an average sequencing depth of 32.60× (Table S6). Notably, only 828 (3.3e-05%) homologous SNPs were detected after mapping the short reads back to the assembled genome, indicating a high level of sequence accuracy (see Table S7). Further analysis revealed that the assembled genome contained 14,874,703 bp of collapsed bases, representing a low collapse rate of 0.58% (detailed in Fig. S2). Moreover, Merqury calculated a Quality Value (QV) of 49.66, reflecting a high genome completeness of 96.51%. Additionally, to evaluate the completeness of the *D. delphis* genome, CEGMA analysis was employed. This analysis identified that 245 out of the 248 core eukaryotic genes (CEGs), accounting for 98.79% completeness, were present in our assembled genome, as shown in Table S8. It is pertinent to note that the term ‘Prots’ in Table S8 denotes these assembled core genes, underscoring the robustness of our genomic assembly in capturing essential protein-coding sequences representative of eukaryotic life. According to the BUSCO analysis, our genome assembly contains 9,226 genes, with a completeness of 91.0% for the genome and 90.0% for the gene model (Table S9, Fig. S3). Additionally, the analysis identified that 41.78% of the genome assembly, or roughly 1,069.10 Mb, is comprised of repeat sequences (Table 1). The majority of these repeats are long terminal repeats (LTRs) and long interspersed nuclear elements (LINEs), constituting 30.72% and 18.96% of the transposable elements, respectively (Table S10).

**Table 2:** Summary of functional annotations for predicted genes

	Number	Percentage (%)
Total	22,148	100
Swiss-Prot	19,204	86.70
Nr	20,320	91.70
KEGG	17,576	79.40
InterPro	21,026	94.90
GO	14,000	63.20
Pfam	16,843	76.00
Annotated	21,364	96.50
Unannotated	784	3.50

The genome assembly predicts a total of 22,148 genes, of which 21,364 (96.50%) have been functionally annotated using various gene databases (Tables 1 and 2). Additionally, we have identified a significant number of non-coding RNAs, including 24,996 miRNAs, 16,963 tRNAs, 252 rRNAs, and 1,874 snRNAs (Table S11). Remarkably, 22,137 of the predicted genes, or 99.95%, are mapped to chromosomes. This comprehensive mapping is depicted in Fig. 1B. The gene density analysis across the 23 chromosomes reveals variation from 5.41 genes/Mb on chromosome 18 to 20.27 genes/Mb on chromosome 19, with an overall average density of 9.22 genes/Mb (Fig. 1B; Table S12).

### Phylogenetic and divergence analyses

**Fig. 2:**
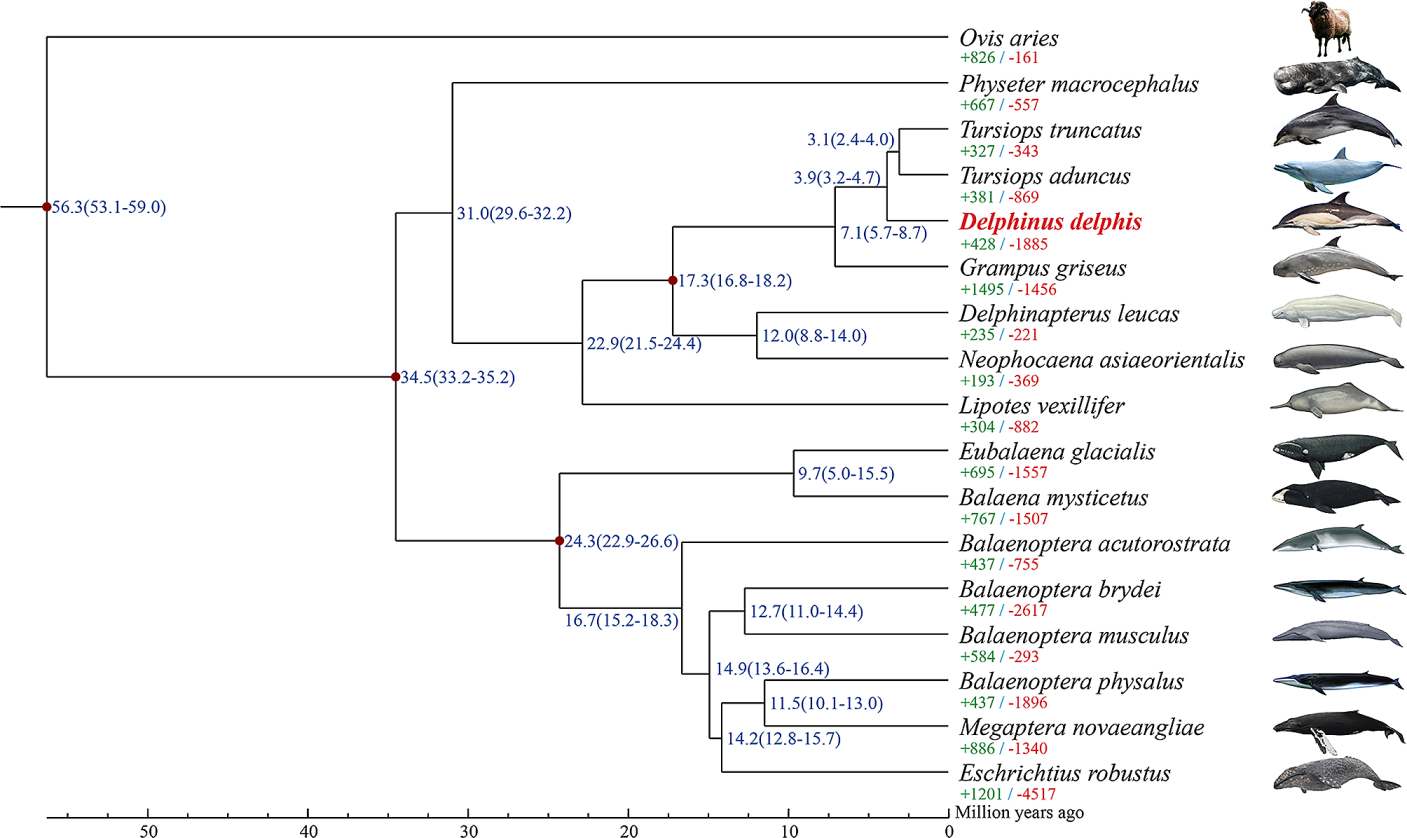
Maximum likelihood phylogenetic analysis of *D. delphis* and other cetaceans with *Ovis aries* outgroup. Each branch site shows the estimated species divergence time (million years ago). The majority of the branches exhibit a branch support of 100. A notable exception is the branching point leading to *Eschrichtius robustus* and the common ancestor of both *B. physalus* and *Megaptera novaeangliae*, which has a branch support of 94. The green numbers indicate the number of gene families that expanded during the evolution of the species and the red numbers indicate the number of gene families that contracted. The sources of species images are provided in Table S1

In this study, we analyzed gene families across 16 whale species and one sheep species, serving as an outgroup, identifying a total of 23,443 gene families. Of these, 5,939 gene families are common to all examined species (Table S13). Notably, *D. delphis* possesses 32 unique gene families, comprising 69 genes that are not found in any other species under study (Table S13). Phylogenetic analysis, based on 2,630 single-copy genes, constructed through maximum likelihood estimation, positions *D. delphis* closely with *T. aduncus* and *T. truncatus*, members of the subfamily Delphininae. Subsequently, these species form a cluster with *G. griseus* of the subfamily Globicephalinae (Fig. 2). The divergence timeline suggests that *D. delphis* and the *Tursiops* species parted ways approximately 3.9 million years ago, in the mid-Pliocene era. Moreover, their common ancestor diverged from *G. griseus* roughly 7.1 million years ago, during the late Miocene period (Fig. 2). This study thus offers an in-depth look into the phylogenetic relationships and divergence timelines within Cetacea (Fig. 2).

### Specific gene family evolution in *D. Delphis*

In our analysis, we identified 428 expanded and 1,885 contracted gene families in *D. delphis* (Fig. 2). Notably, 80 gene families exhibited significant expansion, while 54 showed significant contraction (*p* < 0.01) (Fig. 2). These expansions and contractions of gene families are assessed relative to their common ancestors with adjacent species, including *T. truncatus* and *T. aduncus*. Further analysis through KEGG pathway enrichment revealed that contracted gene families predominantly associate with biological processes such as gap junction, alpha-linolenic acid metabolism, linoleic acid metabolism and phagosome (Fig. S4). In contrast, significantly expanded gene families were involved in the RIG-I-like receptor signaling pathway (ko04622), JAK-STAT signaling pathway, natural killer cell mediated cytotoxicity and cytokine-cytokine receptor interaction, etc. (KEGG, Fig. S5). Among the expanded gene families, three were highlighted for their potential pivotal roles in *D. delphis*’ biological functions, as evidenced by KEGG enrichment analysis (Fig. S5).

**Fig. 3:**
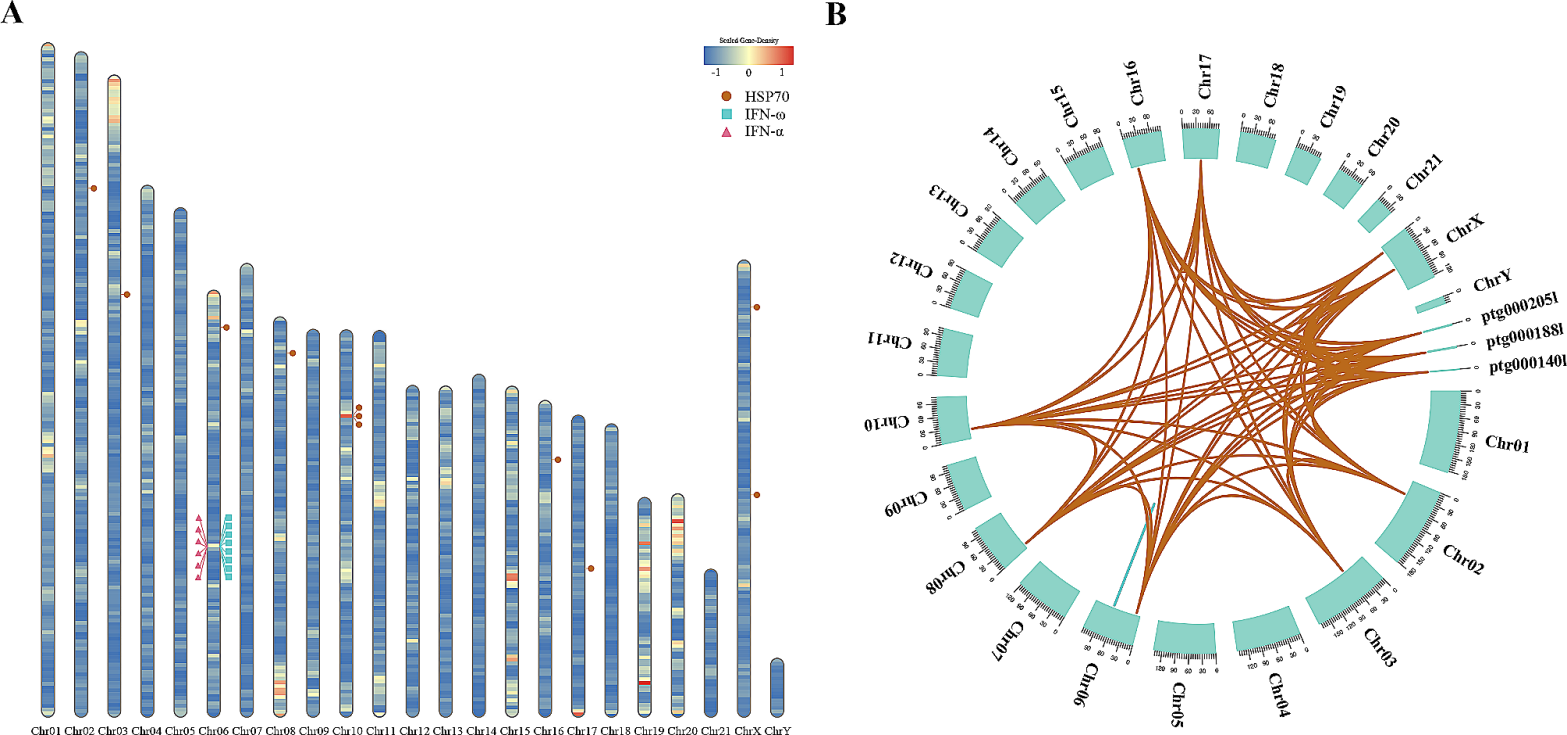
Distributions and sequence identity of three expanded gene families in the genome of *D. delphis*. (**A**) Chromosomal distribution of HSP70, IFN-α, and IFN-ω genes in *D. delphis*, with color-coded gene density (genes/Mb) heat map. (**B**) Sequence identity of the HSP70, IFN-α, and IFN-ω genes in the genome of *D. delphis*

**Fig. 4:**
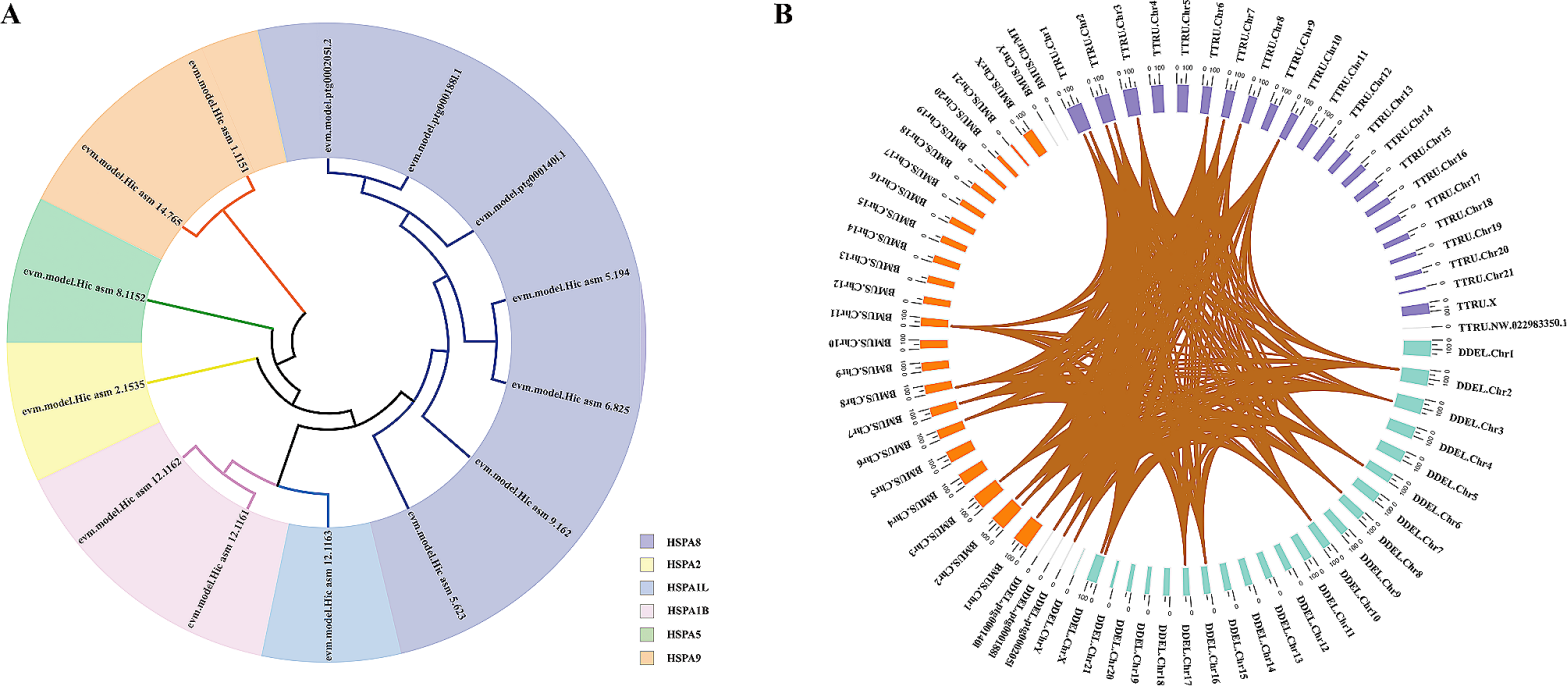
Phylogenetic and synteny analysis of HSP70 genes. (**A**) Maximum likelihood phylogenetic tree constructed using HSP70 genes in *D. delphis*. Gene identifiers, such as “evm.model.Hic asm 9.162”, are used to label specific genes within the HSP70 family. (**B**) Synteny analysis of HSP70 genes among *D. delphis*, *T. truncatus*, and *B. musculus*. The color tags represent species identifiers: blue green for *D. delphis* (DDEL), purple for *T. truncatus* (TTRU), and orange for *B. musculus* (BMUS). Notations like “Chr1” are used to indicate chromosome numbers

We identified 14 HSP70 (K03283, Hsp70) family genes scattered across 8 chromosomes and three contigs within the *D. delphis* genome (Fig. 3A). Abundant pairs of HSP70 gene segmental duplicates were found in the genome (Fig. 3B, Table S14). In contrast to *T. truncatus*, which possesses 5 HSP70 family members, *D. delphis* exhibits a richer diversity with these genes segregating into 6 distinct subgroups. This classification was based on an in-depth phylogenetic analysis and examination of motif compositions (Fig. 4A, Fig. S6). Moreover, the collinearity analysis between *D. delphis* and the two representative species, *T. truncatus* and *B. musculus*, highlighted numerous homologous HSP70 gene pairs present within the syntenic regions (Fig. 4B). Notably, *D. delphis* shows a unique distribution pattern, with half of its HSP70 genes falling within the HSPA8 subgroup (Fig. 4A), a proportion that stands in contrast to *T. truncatus* (40%) and *B. musculus* (22%) (Fig. S6).

**Fig. 5:**
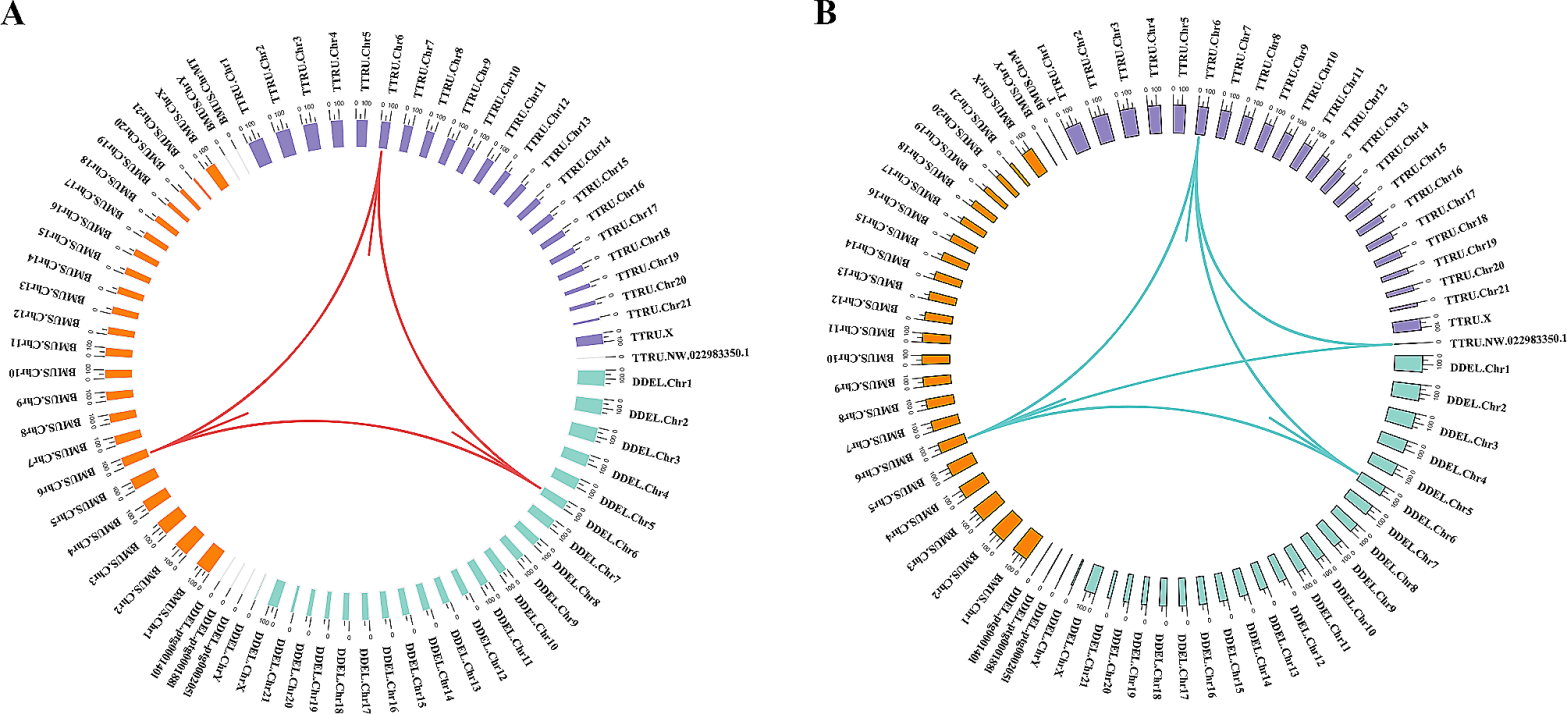
Synteny analysis and expression profiling of IFN-α and IFN-ω genes. (**A**) Synteny analysis of IFN-α genes among *D. delphis*, *T. truncatus*, and (**B**) musculus. The abbreviations “DDEL”, “TTRU”, and “BMUS” represent *D. delphis*, *T. truncatus*, and *B. musculus*, respectively. Notations like “Chr1” are used to indicate chromosome numbers. B. Synteny analysis of IFN-ω genes among the three aforementioned species

According to our results, we discerned a specific clustering of interferon genes within the *D. delphis* genome, locating 6 IFN-α (K05414, IFNA) and 8 IFN-ω (K05440, IFNW) genes primarily on chromosome 6 (Fig. 3A). The genes of the IFN-α and IFN-ω families exhibited fairly high sequence identity (Fig. 3B, Table S15, Table S16). In addition, in the synteny analyses, 3 and 6 pairs of homologous genes from the IFN-α and IFN-ω gene families, respectively, were present within the syntenic regions among the genomes of *D. delphis*, *T. truncatus*, and *B. musculus* (Fig. 5A, B). Further phylogenetic and motif analysis led to the categorization of *D. delphis*’ IFN-α and IFN-ω genes into distinct subgroups: IFN-α1, IFN-α2, IFN-α3 for IFN-α, and IFN-ω1, IFN-ω2 for IFN-ω (Fig. S7, Fig. S8).

### Genes under positive selection

In our study, we identified 120 genes under positive selection, as indicated by a false discovery rate (FDR) of less than 0.05. Through comprehensive KEGG pathway enrichment analysis [81], these genes predominantly contribute to several key biological processes, including histidine metabolism, the FoxO signaling pathway, riboflavin metabolism, the RIG-I-like receptor signaling pathway, sulfur metabolism, ubiquinone and other terpenoid-quinone biosynthesis (Fig. S9). Notably, three of these genes—tripartite motif-containing protein 25 (K10652, TRIM25), peptidyl-prolyl cis-trans isomerase NIMA-interacting 1 (K09578, PIN1), and p38 MAP kinase (K04441, p38)—emerged as critical components of the RIG-I-like receptor signaling pathway, underscoring their potential pivotal roles in adaptive responses.

### Demographic history

**Fig. 6:**
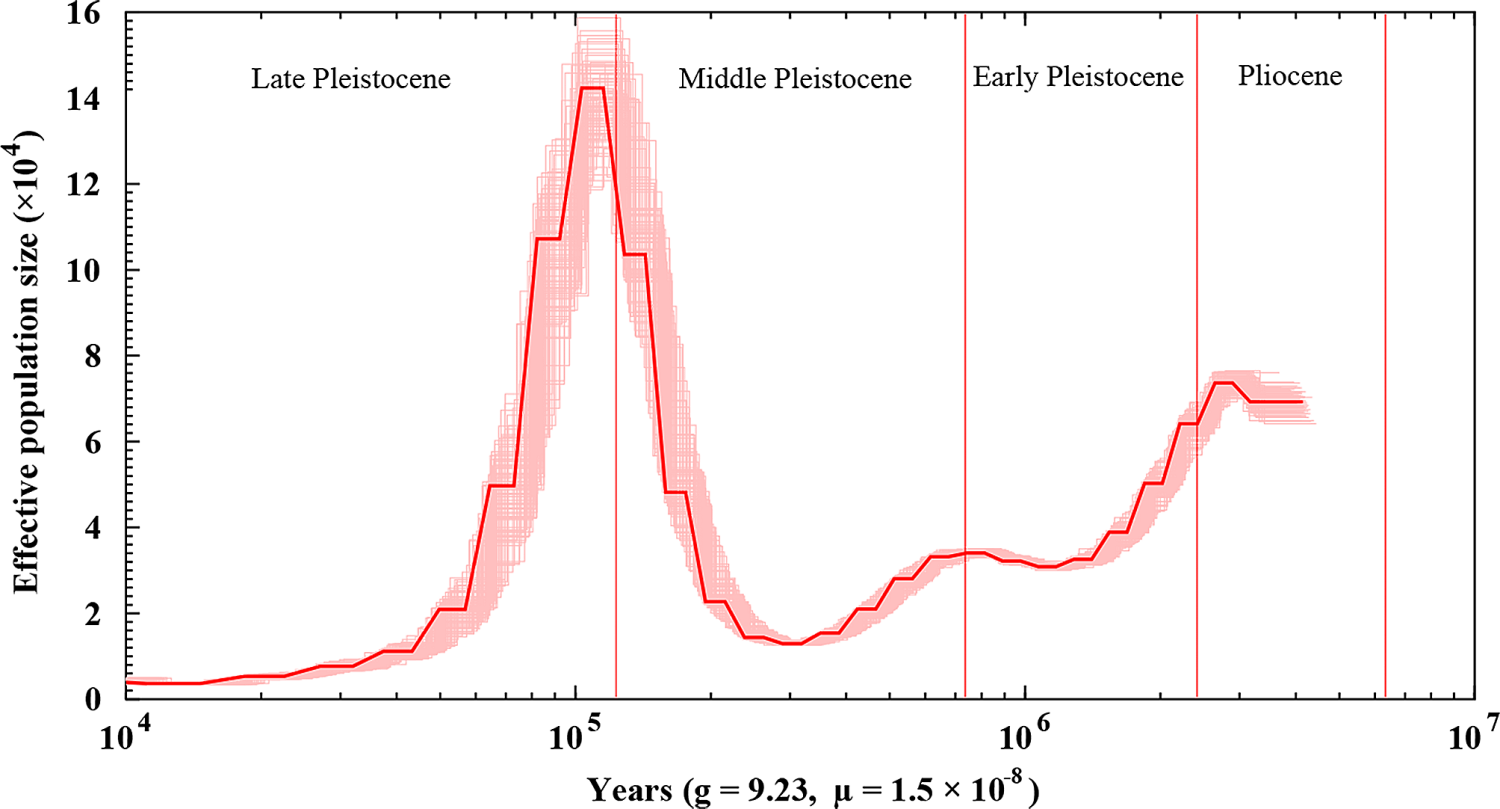
Historical effective population size of *D. delphis* based on PSMC analysis. The thick red line represents the primary PSMC estimate from the original genomic data. The light red lines indicate bootstrap replicates to reflect the variability and robustness of the PSMC estimates

The effective population size (Ne) of *D. delphis* ranged from 0.4 × 10^4^ to 14.2 × 10^4^ during the period of 4000  ∼ 10 Ka (Fig. 6). The PSMC indicated there was a substantial population decrease from 7.5 × 10^4^ to 1.2 × 10^4^ during the early Pleistocene age and the first half of the middle Pleistocene age (320,000–2,430,000 years ago) (Fig. 6). After that, the population of common dolphins increased sharply to its peak in the early upper Pleistocene age (about 120,000 years ago) and then decreased drastically to its minimum at the end of the upper Pleistocene age (about 10,000 years ago) (Fig. 6).

## Discussion

### Quality and significance of *D. Delphis* genome assembly

Our assembly of the *D. delphis* genome showcases outstanding quality, underscored by the minimal homologous SNPs identified upon re-mapping the short reads to the assembled genome, thereby implying a remarkably low error rate. Such precision aligns with the current upward trend in cetacean genome assembly quality [88], marking a significant stride in the field. Notably, the assembly’s Quality Value (QV) stands at an impressive 49.66, highlighting the assembly’s accuracy and the genome’s detailed representation. When compared to the genome of *T. truncatus*, the *D. delphis* assembly not only boasts a greater contig N50 length but also encompasses a wider array of predicted non-coding RNA genes (Table 1). This comparison serves to underline the depth and comprehensiveness of our genomic assembly, illustrating its substantial contribution to cetacean genomics.

The notable synteny observed between the genomes of *D. delphis* and *T. truncatus* underscores the evolutionary stability prevalent within the Delphinidae family (Fig. 1C). This pattern of synteny is not unique to these two species, it has also been identified in the genomes of other cetaceans, including the rough-toothed dolphin (*Steno bredanensis*) and the melon-headed whale (*Peponocephala electra*), underscoring a broader trend of evolutionary conservation among cetacean species [89]. The high-quality genome assembly of *D. delphis* introduced in this research thus emerges as a crucial asset for comparative genomic studies aimed at unraveling the ecological and evolutionary dynamics that shape the Delphinidae family.

### Evolutionary relationships and genomic conservation in *D. Delphis*

The discernment of a modest quantity of gene families exclusive to *D. delphis* indicates an evolutionary distinctiveness, notwithstanding a significant genomic conservation among the cetaceans analyzed. The molecular phylogenetic analysis, showcasing a close kinship between *D. delphis* and the *Tursiops* species (*T. aduncus* and *T. truncatus*), aligns with prior classifications derived from RADseq data [78]. This alignment underscores the efficacy of utilizing diverse genomic methodologies for elucidating cetacean phylogenies. The estimated divergence times, pinpointing the evolutionary separation of *D. delphis* from its nearest kin in the Pliocene and Miocene epochs, shed light on the adaptive and evolutionary trajectories of common dolphins throughout these periods. Additionally, the congruence of our findings with earlier research based on targeted sequence capture [79] reinforces the dependability and consistency of phylogenetic reconstructions in cetaceans.

### Adaptive evolution of key gene families in *D. Delphis*

The expansion and contraction of gene families play pivotal roles in the evolution of adaptive traits [90]. Notably, the pronounced contraction of gene families associated with gap junctions and fatty acid metabolism, coupled with the expansion in gene families involved in signaling and immune response pathways, suggests specific evolutionary pressures and adaptation strategies in *D. delphis*. These observations emphasize the significance of gene family dynamics in unraveling evolutionary paths and potential adaptive strategies. Moreover, the discovery of three specifically expanded gene families, deemed to have substantial functions, points to unique areas of biological significance in *D. delphis*.

Heat shock protein 70 (HSP70) represents a highly conserved gene family with members identified in both prokaryotes and eukaryotes [91–93]. The HSP70 gene family’s high conservation, evidenced by abundant segmental duplicates, indicates its evolutionary importance in common dolphins. Conserved syntenic regions shared among *D. delphis*, *T. truncatus*, and *B. musculus* underline the gene family’s crucial role in cetacean adaptation. Variations in HSP70 members among these species [94] and their gene distribution hint at species-specific evolutionary adaptations. Notably, the HSPA8 subgroup in *D. delphis* suggests specialized cellular stress response mechanisms.

The expansion of the HSP70 gene family in common dolphins likely aids their adaption to environmental changes like hypoxia, temperature shifts, and pH variations. HSP70 genes are vital for cellular protection against stress and promoting cell survival [91, 95, 96]. HSP70 proteins can buffer against diverse cellular stresses, including elevated temperature, pH fluctuations, hypoxia, pollutants, and oxidative stress [93, 97]. This might be related to their broad distribution [1, 2], ability to dive to extreme depths, travel long distances, and highly mobility [4, 7]. Diverse *D. delphis* populations show significant genomic variation in coasts with fluctuating sea conditions [8]. Such genetic traits likely underscore their adaptability to intricate marine habitats.

The expansion of the HSP70 gene family might also contribute to the *D. delphis’* longevity and resistance to accumulated pollutants. Dolphins, notably *D. delphis* with lifespans up to 30 years [6, 14], exhibit exceptional longevity [98]. Cellular stress resistance in animals is correlated with longevity [99], with enhanced resistance being vital for longer-lived species [100, 101]. A strong correlation between HSP70 expression and longer lifespans suggests elevated basal HSP70 levels in longer-lived animals [99]. While a link between longevity and elevated HSP expression is established in various species [99], its specific implication for *D. delphis* remains to be determined. Given cetaceans’ status as marine pollution indicators due to pollutant accumulation [12, 102], HSP70 genes’ potential in mitigating toxic effects is notable [97, 103].

Interferons (IFNs) are potent cytokines that play a crucial role in the process of innate immune responses, which have shown antiviral, antineoplastic, and anti-inflammatory functions [104, 105]. Type I IFNs (including IFN-α, IFN-β, IFN-ω, etc.) are a class of the three IFN subtypes [106, 107]. The genes of the IFN-α and IFN-ω families in the *D. delphis* genome exhibited a pronounced sequence identity. This conservation, further supported by the presence of homologous gene pairs from the IFN-α and IFN-ω gene families within syntenic regions across *D. delphis*, *T. truncatus*, and *B. musculus*, emphasizes the evolutionary significance of these gene families in cetaceans. The observed conservation may suggest a conserved function and mechanism of action for these genes across cetaceans. Given the fundamental role of interferons in innate immune responses, such conservation underscores their importance in mediating antiviral and anti-inflammatory responses in cetaceans [108, 109]. IFN genes may be transcriptionally upregulated in response to specific stimuli, such as immune challenges, potentially accounting for their observed low baseline expressions across the surveyed tissues in this study [110].

The expansions in the IFN-α and IFN-ω gene families in the genome of *D. delphis* may indicate strong antiviral, anti-inflammatory, and antineoplastic abilities in common dolphins. IFN-α and IFN-ω are the two most important type I IFNs, and they have particularly potent antiviral, antiproliferative, anti-inflammatory, and immunomodulatory properties [108, 109]. IFN-α is a key cytokine in the innate immune response induced by infections and tissue stress and damage [104]. It is generated by fibroblasts and T- and NK-cells [111] to combat unrecognized organisms and cells, including viruses and tumor cells [104]. Clinically, IFN-α is used to prevent or treat different viral infections [112, 113] and it also produces beneficial effects in several tumor-associated diseases [114, 115]. In addition, IFN-ω is secreted primarily by virus-infected leukocytes and has been identified in numerous mammalian groups [116, 117]. It activates the phosphatidylinositol-3-kinase/protein kinase B (P13K/Akt) signaling pathway to up-regulate antiviral activity [118, 119]. Compared with IFN-α, IFN-ω is more effective in inhibiting virus replication and has exhibited some degree of cross-species activity [120, 121]. Furthermore, it has been shown to have anti-proliferation and antitumor effects [107].

### Implications of genes under positive selection in immune and antitumor mechanisms

Positively selected genes often reflect evolutionary adaptations to specific environmental challenges or physiological needs [122]. In the context of our study, three such genes emerged as particularly intriguing due to their involvement in the RIG-I-like receptor signaling pathway, which is pivotal for the recognition of viral pathogens and the modulation of innate immune responses. The first, TRIM25, is an E3 ubiquitin ligase enzyme that regulates the K63-linked ubiquitination of RIG-I, which is essential for RIG-I downstream signaling and the innate immune response to viral infections [123]. TRIM25 also modulates cell proliferation and migration [124, 125], apoptosis, and plays a key role in tumorigenesis [126, 127]. Previous studies revealed that TRIM25 is under positive selection pressure in primates [128, 129]. The second, PIN1, is a member of the parvulin family that modulates a large subset of key oncogenes and tumor suppressors by catalyzing the cis/trans isomerization of specific pSer/Thr-Pro motifs [130, 131]. PIN1 facilitates the functions of multiple oncogenes and abrogates tumor suppressors [132], and hence its deregulation results in disease, especially cancer [133]. Humans with genetic polymorphisms that reduce PIN1 expression have a lower risk for multiple cancers [134, 135]. Similarly, PIN1 knockout mice are highly resistant to tumorigenesis, even amid the overexpression of oncogenes [136, 137]. Besides, PIN1 also plays a role in inflammatory diseases and viral infections [130]. The third, p38, is one of the four main subgroups of mitogen-activated protein kinases (MAPKs) [138]. It is involved in numerous complex biological processes, including cell proliferation, cell differentiation, cell death, cell apoptosis, cell migration, and cell invasion [138, 139]. Its pathways can be activated in response to a variety of environmental and cellular stresses, such as inflammation and tumorigenesis, among other signals [140, 141].

According to the properties and functions of these three genes in the RIG-I-like receptor signaling pathway, which are under positive selection pressures, they may also be related to the anti-inflammatory and anti-tumor mechanisms [142] in *D. delphis*. As cetaceans are a long-lived class of mammals, they should have already developed antitumor mechanisms [143]. A variety of tumor suppressor genes (TSGs) have been investigated in cetaceans before, and their positive selection and gene duplication provided some insight into how cetaceans resist cancers [143]. Many TSGs are involved in cellular proliferation, differentiation, and apoptosis [144], and are related to diseases, especially cancers [145, 146].

### Historical population dynamics and influencing factors in *D. Delphis*

The estimation of the historical effective population size is intrinsically tied to our understanding of the species’ evolutionary history [147]. As it provides insights into the past population size of the species and how historical events, such as environmental changes, hybridization, migration, or disease outbreaks, have impacted the population [148]. Compared with other cetaceans such as *T. truncatus*, *T. aduncus*, *B. acutorostrata*, etc [78, 149].., the population of *D. delphis* experienced larger (or abnormal) contractions and expansions during the Pleistocene. This divergence in demographic history is consistent with our phylogenetic findings, suggesting that *D. delphis*, *T. truncatus*, and *T. aduncus* have a common ancestor but have experienced distinct evolutionary trajectories. Our genomic data supports the notion that these population dynamics in *D. delphis* are not only unique but also a key component in understanding its evolutionary history. One previous study estimated that *D. delphis* originated in the Pacific Ocean of the Northern Hemisphere [150], and a cooling of the tropical Pacific during the Pleistocene may have driven them to disperse across equatorial waters to the Southern Hemisphere [151, 152]. Simultaneously, new niches would be opened due to the changes in primary productivity and prey abundance [153], which might have facilitated the colonization of common dolphins around the world. Indeed, there were rapid climatic changes and oceanographic shifts during the mid-Pleistocene, and the subsequent stage of high productivity in the Pacific Ocean, owed to a major cooling event [152, 154], might have led to a drastic increase of Ne in this species. The rapid reduction in Ne that followed might have resulted from a subsequent decrease in productivity due to higher temperatures [150]. On the other hand, common dolphins are known to hybridize with other dolphins [155, 156], hence the dramatic fluctuations in their effective population size estimated by genomic data might also be induced by substantial genetic exchange with *D. delphis* through hybridization during the Pleistocene [149].

## Conclusions

Here, we presented a high-quality genome of the common dolphin *D. delphis* with 2.56 Gb and 93.81% of contigs anchored onto 23 chromosomes. Genomic comparisons showed that *D. delphis* is closely related to *T. truncatus* and *T. aduncus*, and diverged ca. 3.9 MYA. The expansions of the HSP70, IFN-α, and IFN-ω gene families, and the positively selected genes encoding TRIM 25, PIN1, and p38, might underlie the evolutionary success of common dolphins. These data also indicated drastic contractions and expansions of the effective population size of *D. delphis* during the Pleistocene. This high-quality genome data represents significant new resources for cetacean and mammalian studies.

### Electronic supplementary material

Below is the link to the electronic supplementary material.

Supplementary Material 1

Supplementary Material 2

Supplementary Material 3

## Data Availability

The datasets presented in this study are available in the NCBI Sequence Read Archive under the accession number PRJNA903213, https://www.ncbi.nlm.nih.gov/datasets/taxonomy/9728/.
