# Correction: Chromosome-level genome provides insights into environmental adaptability and innate immunity in the common dolphin (*Delphinus delphis*)

**DOI:** 10.1186/s12864-024-10371-6

**Published:** 2024-05-21

**Authors:** Kui Ding, Qinzeng Xu, Liyuan Zhao, Yixuan Li, Zhong Li, Wenge Shi, Qianhui Zeng, Xianyan Wang, Xuelei Zhang

**Affiliations:** 1 Key Laboratory of Marine Eco-Environmental Science and Technology, First Institute of Oceanography, Ministry of Natural Resources, Qingdao, China; 2 Laboratory for Marine Ecology and Environmental Science, Qingdao Marine Science and Technology Center, Qingdao, China; 3 Key Laboratory of Marine Ecological Conservation and Restoration, Third Institute of Oceanography, Ministry of Natural Resources, Xiamen, China; 4 National Engineering Laboratory for Integrated Aero-Space-GroundOcean Big Data Application Technology, Xi’an, China

**Correction**: *BMC Genomics***25**, 373 (2024). 10.1186/s12864-024-10268-4.

Following publication of the original article it was reported that there was a missing co-first authorship note for Kui Ding, Qinzeng Xu, and Liyuan Zhao.

There was also a typographical error in the article title, namely that ‘Delphinus’ was not capitalized.

The original article has been updated.

