# Supplementary material for: Chromosome-level genome provides insights into environmental adaptability and innate immunity in the common dolphin (Delphinus delphis)
Source: BMC Genomics. 2024 Apr 16;25:373. doi: 10.1186/s12864-024-10268-4 (PMC11022445; doi:10.1186/s12864-024-10268-4)
Supplement: Supplementary file 1 — Supplementary Material 1 [file 12864_2024_10268_MOESM1_ESM.docx]

## Supplementary materials and methods

### **Library construction and sequencing**

The process for constructing single molecule real-time (SMRT) PacBio genome sequencing libraries was as follows: high molecule genomic DNA was first randomly fragmented into ~20 kb target sizes, after which damage and end repair, blunt-end adaptor ligation, and size selection were conducted. Finally, the constructed libraries were sequenced on the PacBio Sequel II platform (California, USA).

As for the construction of Hi-C library of *D. delphis* muscle tissues, genomic DNA in muscle tissue was first fixed with paraformaldehyde, then homogenized in tissue lysis buffer, treated with the restriction endonuclease MboI, marked with biotin, proximity ligated to generate chimeric junctions, and finally purified and fragmented. This entire procedure was conducted in-house and did not utilize any commercial kits. The Hi-C library was then sequenced on the Illumina NovaSeq 6000 platform (USA).

RNA from the five main tissues was sequenced to assist genome annotation.The mRNAs that contained a polyA tail were enriched from the total RNA using the magnetic beads method, then divalent cations were used in the NEB fragmentation buffer. The obtained mRNAs were randomly fragmented, and the library was constructed using the NEB method of library construction. The NEBNext® Ultra™ RNA Library Prep Kit for Illumina® (USA) was used to construct the library according to the manufacturer’s instructions. Qubit was used for preliminary quantification, then the library was diluted to 1.5 ng/μl. Agilent 2100 was used to detect the library’s fragment lengths, and qRT-PCR was used to quantify the library’s effective concentration (> 2 ng/μl). Finally, the constructed library was sequenced by PE150 on the Illumina Hiseq platform (USA). All genomic DNA and RNA sequencing were conducted by Novogene Co., LTD (Beijing, China).

### **Gene family, phylogenetic analysis, and divergence time estimation**

TBtools [1] was used to filter any repeat annotations in each species. Representative coding regions (CDS) and peptide sequences were extracted from the genome according to the filtered annotations for downstream analysis. We used Orthofinder [2] to cluster peptide sequences for single copy orthologous genes. Gene families (the orthologous groups) were collected from these 17 species according to the gene clustering results.

The protein sequences of single-copy gene families were selected and used in multiple sequence alignment by MAFFT v7.487 [3], and the results were then reversed to the CDS multiple sequence alignment. Three super alignment matrixes were constructed by combining the alignment results of all single-copy genes in three ways: (a) directly linking the alignment results of all single-copy genes; (b) combining the alignment results of all single-copy genes by ligation, and using Gblocks v0.91b [4] to extract conserved sequences; and (c) conserved sequences were extracted from the alignment results of each single-copy gene by Gblocks, and then the conserved sequences were linked. All untreated sites, phase1 sites, and 4D sites (quadruple degenerate sites) were obtained from each super alignment matrix. A total of 9 sets of data were acquired and each set was used to construct a phylogenetic tree in RAxML v8.2.12 [5] with the maximum likelihood (ML) method. Ultimately, the final phylogenetic relationships were determined based on the known relationships among species and the degree of agreement among the 9 phylogenetic trees.

Based on the sequences of the single-copy gene families, the divergence time was estimated using mcmctree v4.9 in the PAML software package [6]. The specific methods were as follows: (a) 4D sites were extracted from the single-copy genes; (b) the divergence time of related species was obtained using the Timetree website; (c) mcmctree was used to estimate the gradient and Hessian parameters; and (d) the correlated molecular clock and JC69 models were selected to estimate divergence times.

### **Detection of positively selected genes**

Genes from the single-copy gene families were used in the search for PSGs. We used MAFFT v7.487 [3] to perform multiple sequence alignment on the protein sequences in each single-copy gene family, and then the results were reversed to the CDS multiple sequence alignment. During calibration, *D. delphis* was set as the foreground branch, and the other 16 species were used as background branches. The likelihood values were calculated based on Model A (which assumes that the foreground branch ω undergoes positive selection, that is, ω > 1) and null mode (does not allow the ω of any site value to be greater than 1, that is, ω ≤ 1) separately. Likelihood ratio tests (LRTs) were performed on the above likelihood values using the chi2 program of PAML [6], and significant differences were obtained after *p*-value correction (false discover rate (FDR) < 0.05). The Bayes empirical Bayes method (BEB) was used to obtain the posterior probability of the positively selected sites (usually > 0.95 is considered as significantly positively selected). In addition, GO and KEGG enrichment analyses were performed using Fisher’s exact test with FDR < 0.05.

### **Collinearity analysis**

The complete protein sequences and annotation of *D. delphis* and *T. truncatus* were taken as the input files, and the respective databases of these two species were made using their protein sequences. Then the two databases were blasted to each other with an E-value of 1e-10. The genes with greater than 65% identity were selected to examine collinear relationships between the two species. The chromosome scale collinearity analysis between the common dolphin and bottlenose dolphin genomes was conducted to reveal the evolutionary processes of chromosomes in cetaceans.

1. Sambrook JAR, Russel DW. Molecular cloning. 2001.

2. Liu B, Shi Y, Yuan J, Hu X, Zhang H, Li N, et al. Estimation of genomic characteristics by analyzing k-mer frequency in de novo genome projects. 2013. p. 62-7.

3. Guillaume, Mar?ais, Carl, Kingsford. A fast, lock-free approach for efficient parallel counting of occurrences of k-mers. Bioinformatics. 2011.

4. Luo R, Liu B, Xie Y, Li Z, Liu Y. SOAPdenovo2: an empirically improved memory-efficient short-read de novo assembler. GigaScience. 2012;1.

5. Myers G. Efficient Local Alignment Discovery amongst Noisy Long Reads. ALGORITHMS IN BIOINFORMATICS. 2014:52-67.

6. Zhou R, Li S, Yao W, Xie C, Li K. The Meishan pig genome reveals structural variation mediated gene expression and phenotypic divergence underlying Asian pig domestication. Molecular Ecology Resources. 2021.

7. Chin C-S, Peluso P, Sedlazeck FJ, Nattestad M, Concepcion GT, Clum A, et al. Phased diploid genome assembly with single-molecule real-time sequencing. Nature methods. 2016;13(12):1050-4.

8. Chin C-S, Alexander DH, Marks P, Klammer AA, Drake J, Heiner C, et al. Nonhybrid, finished microbial genome assemblies from long-read SMRT sequencing data. Nature methods. 2013;10(6):563-9.

9. Li H, Durbin R. Inference of human population history from individual whole-genome sequences. Nature. 2011;475(7355):493.

10. Walker BJ, Abeel T, Shea T, Priest M, Earl AM. Pilon: An Integrated Tool for Comprehensive Microbial Variant Detection and Genome Assembly Improvement. PLoS ONE. 2014;9(11):e112963.

11. Bolger AM, Lohse M, Usadel B. Trimmomatic: a flexible trimmer for Illumina sequence data. Bioinformatics. 2014;30(15):2114-20.

12. Li H, Richard D. Fast and accurate short read alignment with Burrows–Wheeler transform. Bioinformatics. 2010(14):14.

13. Blanco E, Parra G, Guigó R. Using geneid to identify genes. Current protocols in bioinformatics. 2007;18(1):4.3. 1-4.3. 28.

14. Manni1 M, Berkeley1 MR, Seppey1 M, Simão1 FA, Zdobnov1CA1 EM. BUSCO update: novel and streamlined workflows along with broader and deeper phylogenetic coverage for scoring of eukaryotic, prokaryotic, and viral genomes. Molecular Biology and Evolution. 2021(No.10):4647-54.

15. Benson G. Tandem repeats finder: a program to analyze DNA sequences. Nucleic acids research. 1999;27(2):573-80.

16. Tarailo‐Graovac M, Chen N. Using RepeatMasker to Identify Repetitive Elements in Genomic Sequences. Current Protocols in Bioinformatics. 2009;25(1).

17. Bao W, Kojima KK, Kohany O. Repbase Update, a database of repetitive elements in eukaryotic genomes. Mobile Dna. 2015;6:1-6.

18. Zhao X, Hao W. LTR_FINDER: an efficient tool for the prediction of full-length LTR retrotransposons. Nucleic Acids Research. 2007;35(Web Server issue):W265-8.

19. Price AL, Jones NC, Pevzner PA. De novo identification of repeat families in large genomes. Bioinformatics. 2005(suppl_1):i351.

20. Stanke M, Keller O, Gunduz I, Hayes A, Waack S, Morgenstern B. AUGUSTUS: ab initio prediction of alternative transcripts. Nucleic acids research. 2006;34(suppl_2):W435-W9.

21. Burge C, Karlin S. Prediction of complete gene structures in human genomic DNA. Journal of molecular biology. 1997;268(1):78-94.

22. Majoros W, Pertea M, Salzberg S. TigrScan and GlimmerHMM: two open source ab initio eukaryotic gene-finders. Bioinformatics. 2004;20(16):2878-9.

23. Altschul SF, Gish W, Miller W, Myers EW, Lipman DJ. Basic local alignment search tool. Journal of molecular biology. 1990;215(3):403-10.

24. Birney E, Clamp M, Durbin R. GeneWise and genomewise. Genome research. 2004;14(5):988-95.

25. Pertea M, Kim D, Pertea GM, Leek JT, Salzberg SL. Transcript-level expression analysis of RNA-seq experiments with HISAT, StringTie and Ballgown. Nature Protocols. 2016;11(9):1650-67.

26. Ghosh S, Chan C-KK. Analysis of RNA-Seq data using TopHat and Cufflinks. Plant Bioinformatics: Methods and Protocols. 2016:339-61.

27. Scott MG, Madden TL. BLAST: at the core of a powerful and diverse set of sequence analysis tools. Nucleic Acids Research. (suppl_2):W20.

28. Jones P, Binns D, Chang H-Y, Fraser M, Li W, McAnulla C, et al. InterProScan 5: genome-scale protein function classification. Bioinformatics. 2014;30(9):1236-40.

29. Hulo N, Bairoch A, Bulliard V, Cerutti L, De Castro E, Langendijk-Genevaux PS, et al. The PROSITE database. Nucleic acids research. 2006;34(suppl_1):D227-D30.

30. Finn RD, Jaina M, John T, Penny C, Andreas H, Pollington JE, et al. The Pfam Protein Families Database. Nucleic Acids Research. 2000;28(1).

31. Attwood TK, Bradley P, Flower DR, Gaulton A, Maudling N, Mitchell AL, et al. PRINTS and its automatic supplement, prePRINTS. Nucleic acids research. 2003;31(1):400-2.

32. Mi H, Betty LU, Rozina L, Anish K, Jody V, Steven R, et al. The PANTHER database of protein families, subfamilies, functions and pathways. Nucleic Acids Research. 2005(suppl_1):D284.

33. Ponting CP, Jrg S, Frank M, Peer B. SMART: identification and annotation of domains from signalling and extracellular protein sequences. Nucleic Acids Research. 1999(1):229-32.

34. Ashburner M, Ball CA, Blake JA, Botstein D, Butler H, Cherry JM, et al. Gene ontology: tool for the unification of biology. Nature genetics. 2000;25(1):25-9.

35. Bru C, Courcelle E, Carrère S, Beausse Y, Dalmar S, Kahn D. The ProDom database of protein domain families: more emphasis on 3D. Nucleic acids research. 2005;33(suppl_1):D212-D5.

36. Uniprot. Reorganizing the protein space at the Universal Protein Resource (UniProt). Nucleic Acids Research. 2012(Suppl):D71-D5.

37. Pruitt KD, Tatiana T, Brown GR, Maglott DR. NCBI Reference Sequences (RefSeq): current status, new features and genome annotation policy. Nucleic Acids Research. 2012(D1):D130-D5.

38. Kanehisa M, Goto S. KEGG: kyoto encyclopedia of genes and genomes. Nucleic acids research. 2000(No.1):27-30.

39. Schattner P, Brooks AN, Lowe TM. The tRNAscan-SE, snoscan and snoGPS web servers for the detection of tRNAs and snoRNAs. Nucleic Acids Research. 2005;33(Web Server issue):686-9.

40. Nawrocki EP, +, Burge SW, +, Bateman A, Daub J, Eberhardt RY, Eddy SR, et al. Rfam 12.0: updates to the RNA families database. Nucleic Acids Research. 2015(D1):D130-D7.

41. Nawrocki EP, Eddy SR. Infernal 1.1: 100-fold faster RNA homology searches. Bioinformatics. 2013(22):2933-5.

42. Chen C, Chen H, Zhang Y, Thomas HR, Frank MH, He Y, et al. TBtools: an integrative toolkit developed for interactive analyses of big biological data. Molecular plant. 2020;13(8):1194-202.

43. Emms DM, Kelly S. OrthoFinder: phylogenetic orthology inference for comparative genomics. Genome biology. 2019;20:1-14.

44. Nakamura, Tsukasa, Yamada, Kazunori, D., Tomii, et al. Parallelization of MAFFT for large-scale multiple sequence alignments. Bioinformatics. 2018.

45. G T, J C. Improvement of phylogenies after removing divergent and ambiguously aligned blocks from protein sequence alignments. Systematic Biology; Washington ?? 2007(No.4):564-77.

46. Alexandros S. RAxML version 8: a tool for phylogenetic analysis and post-analysis of large phylogenies. Bioinformatics. 2014(9):1312-3.

47. Yang Z. PAML 4: Phylogenetic Analysis by Maximum Likelihood. Molecular Biology and Evolution. 2007;24(8):1586-91.

**References**

1. Sambrook JAR, Russel DW. Molecular cloning. 2001.

2. Chen C, Chen H, Zhang Y, Thomas HR, Frank MH, He Y, et al. TBtools: an integrative toolkit developed for interactive analyses of big biological data. Molecular plant. 2020;13(8):1194-202.

3. Emms DM, Kelly S. OrthoFinder: phylogenetic orthology inference for comparative genomics. Genome biology. 2019;20:1-14.

4. Nakamura, Tsukasa, Yamada, Kazunori, D., Tomii, et al. Parallelization of MAFFT for large-scale multiple sequence alignments. Bioinformatics. 2018.

5. G T, J C. Improvement of phylogenies after removing divergent and ambiguously aligned blocks from protein sequence alignments. Systematic Biology; Washington ?? 2007(No.4):564-77.

6. Alexandros S. RAxML version 8: a tool for phylogenetic analysis and post-analysis of large phylogenies. Bioinformatics. 2014(9):1312-3.

7. Yang Z. PAML 4: Phylogenetic Analysis by Maximum Likelihood. Molecular Biology and Evolution. 2007;24(8):1586-91.

1. Sambrook JAR, Russel DW: Molecular cloning. 2001.

2. Chen C, Chen H, Zhang Y, Thomas HR, Frank MH, He Y, Xia R: TBtools: an integrative toolkit developed for interactive analyses of big biological data. Molecular plant 2020, 13(8):1194-1202.

3. Emms DM, Kelly S: OrthoFinder: phylogenetic orthology inference for comparative genomics. Genome biology 2019, 20:1-14.

4. Nakamura, Tsukasa, Yamada, Kazunori, D., Tomii, Kentaro, Katoh, Kazutaka: Parallelization of MAFFT for large-scale multiple sequence alignments. Bioinformatics 2018.

5. G T, J C: Improvement of phylogenies after removing divergent and ambiguously aligned blocks from protein sequence alignments. Systematic Biology; Washington ?? 2007(No.4):564-577.

6. Alexandros S: RAxML version 8: a tool for phylogenetic analysis and post-analysis of large phylogenies. Bioinformatics 2014(9):1312-1313.

7. Yang Z: PAML 4: Phylogenetic Analysis by Maximum Likelihood. Molecular Biology and Evolution 2007, 24(8):1586-1591.

1. Chen C, Chen H, Zhang Y, Thomas HR, Frank MH, He Y, et al. TBtools: an integrative toolkit developed for interactive analyses of big biological data. Molecular plant. 2020;13(8):1194-202.

2. Emms DM, Kelly S. OrthoFinder: phylogenetic orthology inference for comparative genomics. Genome biology. 2019;20:1-14.

3. Nakamura, Tsukasa, Yamada, Kazunori, D., Tomii, et al. Parallelization of MAFFT for large-scale multiple sequence alignments. Bioinformatics. 2018.

4. G T, J C. Improvement of phylogenies after removing divergent and ambiguously aligned blocks from protein sequence alignments. Systematic Biology; Washington ?? 2007(No.4):564-77.

5. Alexandros S. RAxML version 8: a tool for phylogenetic analysis and post-analysis of large phylogenies. Bioinformatics. 2014(9):1312-3.

6. Yang Z. PAML 4: Phylogenetic Analysis by Maximum Likelihood. Molecular Biology and Evolution. 2007;24(8):1586-91.

[1]. Chen C, Chen H, Zhang Y, Thomas HR, Frank MH, He Y, Xia R: TBtools: an integrative toolkit developed for interactive analyses of big biological data. Molecular plant 2020, 13(8):1194-1202.
[2]. Emms DM, Kelly S: OrthoFinder: phylogenetic orthology inference for comparative genomics. Genome biology 2019, 20:1-14.
[3]. Nakamura, Tsukasa, Yamada, Kazunori, D., Tomii, Kentaro, Katoh, Kazutaka: Parallelization of MAFFT for large-scale multiple sequence alignments. Bioinformatics 2018.
[4]. G T, J C: Improvement of phylogenies after removing divergent and ambiguously aligned blocks from protein sequence alignments. Systematic Biology; Washington ?? 2007(No.4):564-577.
[5]. Alexandros S: RAxML version 8: a tool for phylogenetic analysis and post-analysis of large phylogenies. Bioinformatics 2014(9):1312-1313.
[6]. Yang Z: PAML 4: Phylogenetic Analysis by Maximum Likelihood. Molecular Biology and Evolution 2007, 24(8):1586-1591.
