# Supplementary material for: Chromosome-level genome provides insights into environmental adaptability and innate immunity in the common dolphin (Delphinus delphis)
Source: BMC Genomics. 2024 Apr 16;25:373. doi: 10.1186/s12864-024-10268-4 (PMC11022445; doi:10.1186/s12864-024-10268-4)
Supplement: Supplementary file 2 — Supplementary Material 2 [file 12864_2024_10268_MOESM2_ESM.docx]

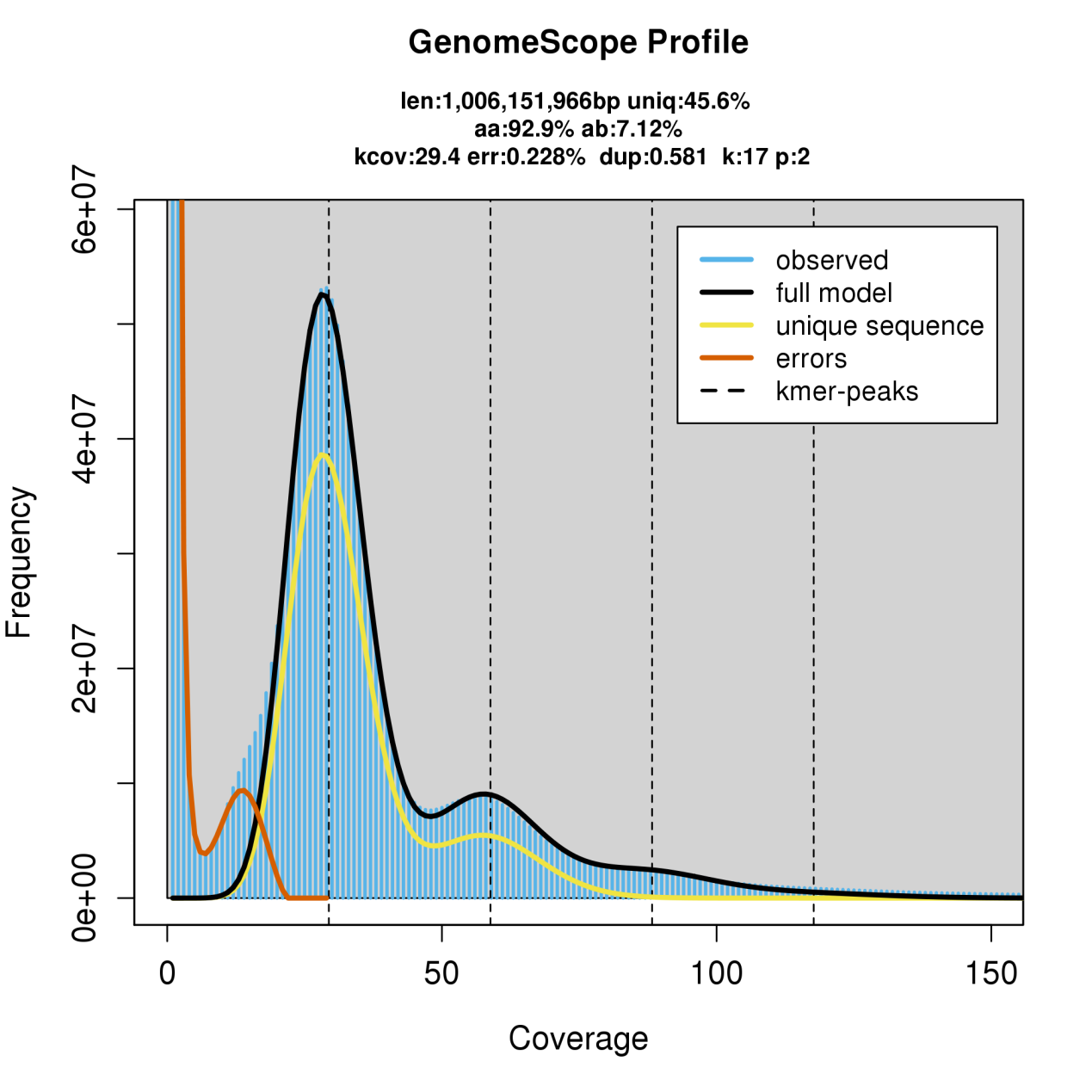


**Fig. S1 Estimation of the genome size of the common dolphin (*D. delphis*) based on GenomeScope analysis. The primary peak value is around a depth of 29.4, and using the formula Kmer-number/depth, the estimated genome size of the dolphin is calculated to be 2,558,368,849 bp.**


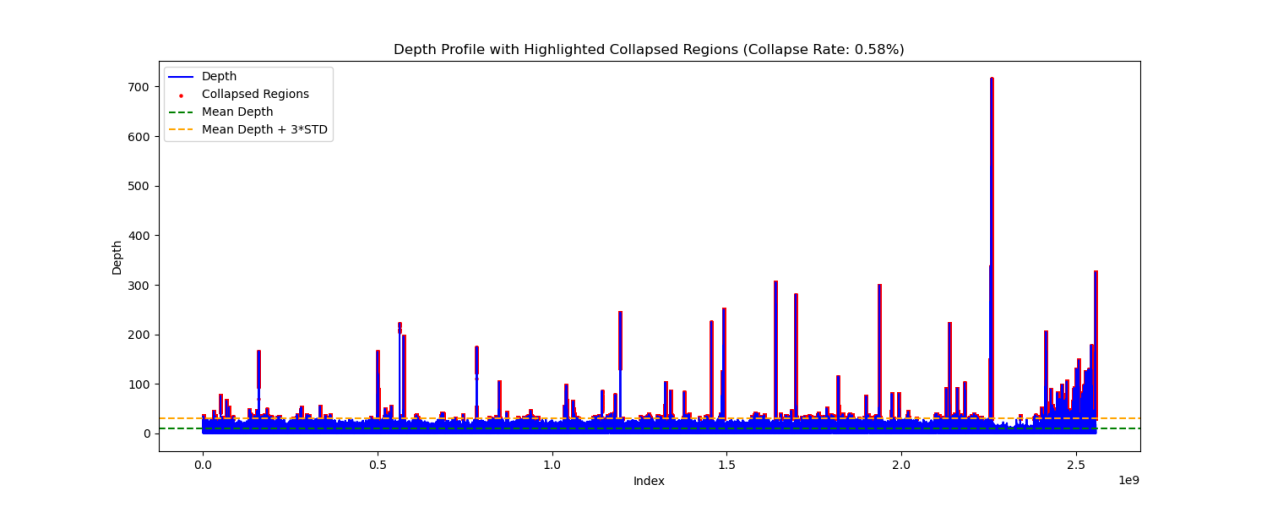


**Fig. S2 Whole genome depth analysis of the common dolphin (*D. delphis*) with highlighted collapsed regions (collapse rate: 0.58%).**


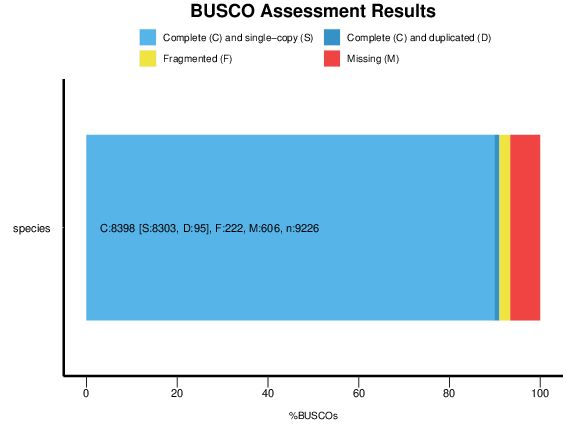


**Fig. S3 BUSCO notation assessment results of *D. delphis* genome**

**
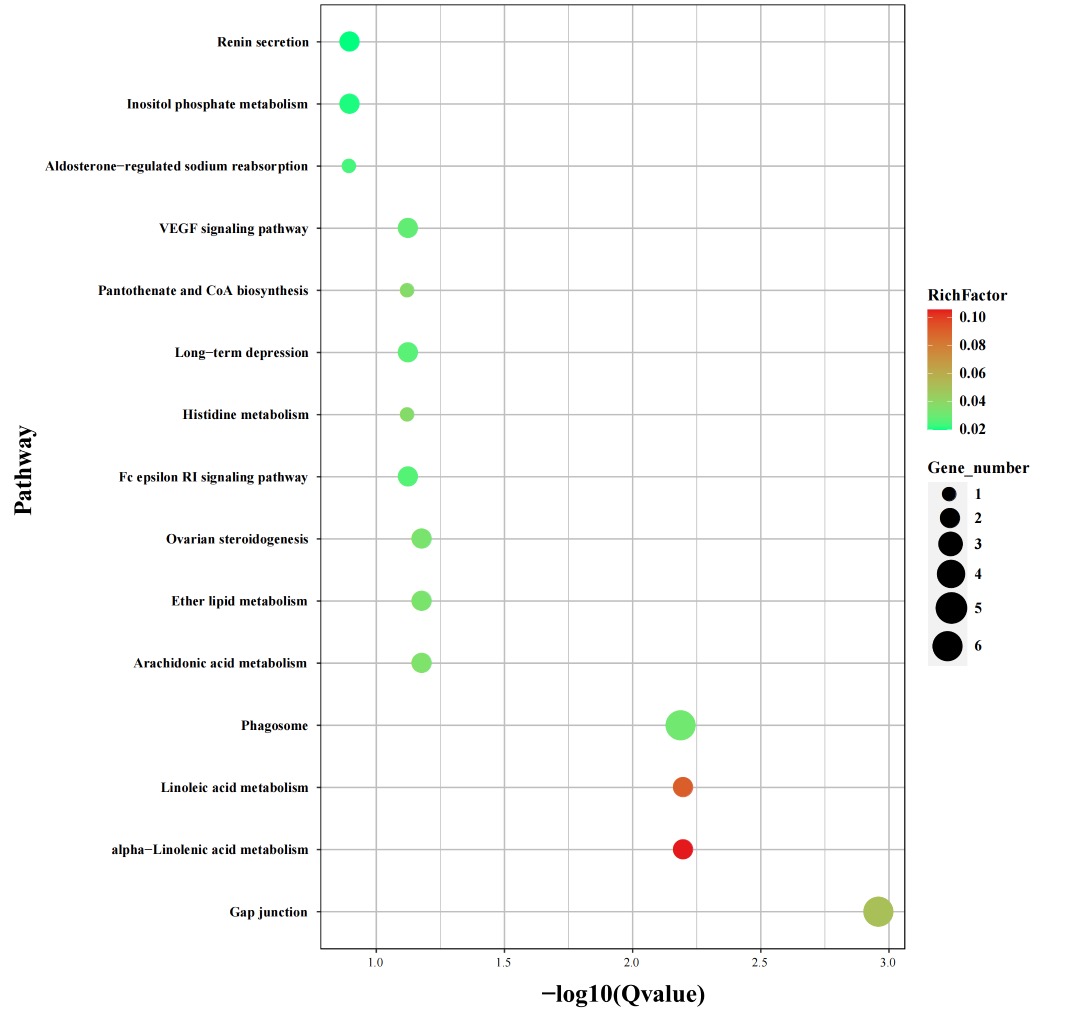
**

**Fig. S4  KEGG enrichment analysis of highly significantly contracted gene families in *D. delphis* (*p* < 0.01).**


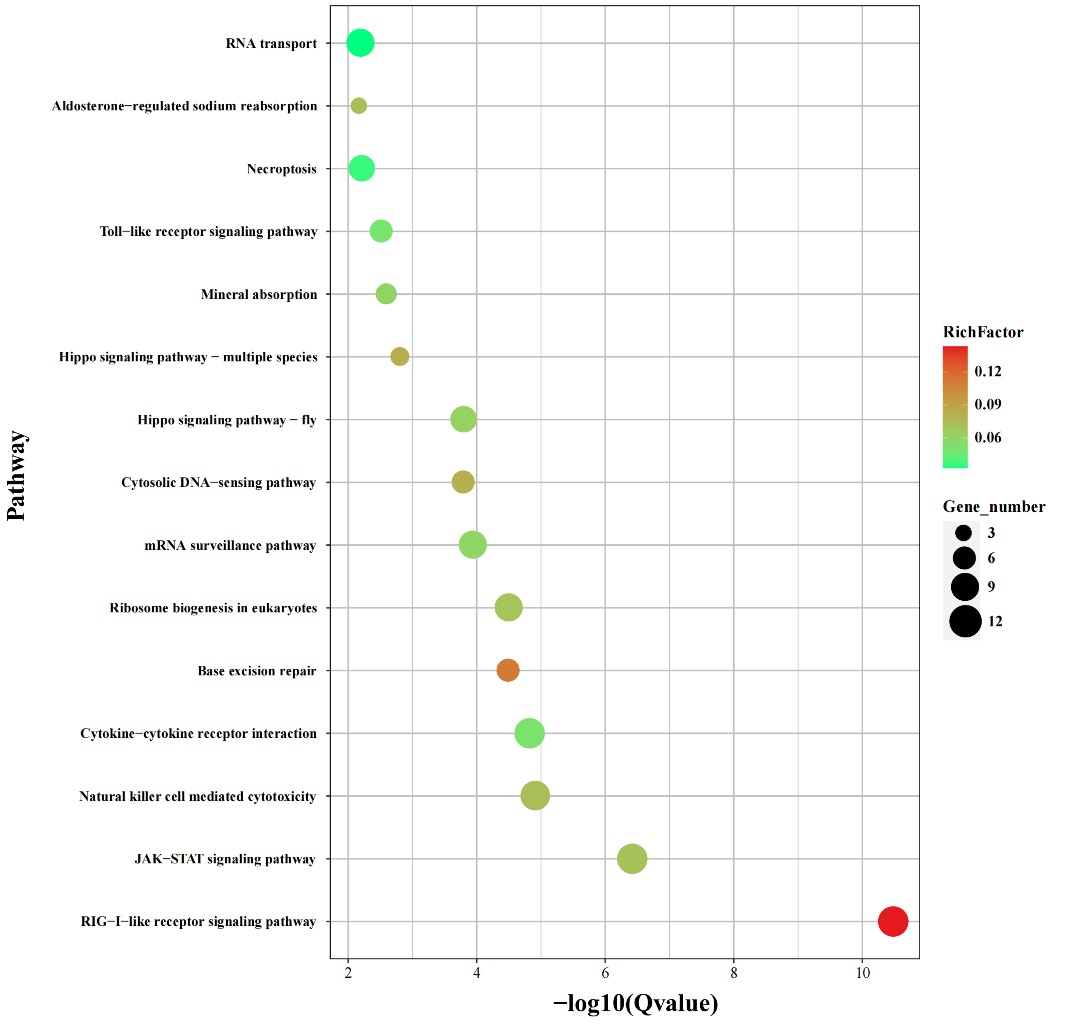


**Fig. S5  KEGG enrichment analysis of highly significantly expanded gene families in *D. delphis* (*p* < 0.01).**

*
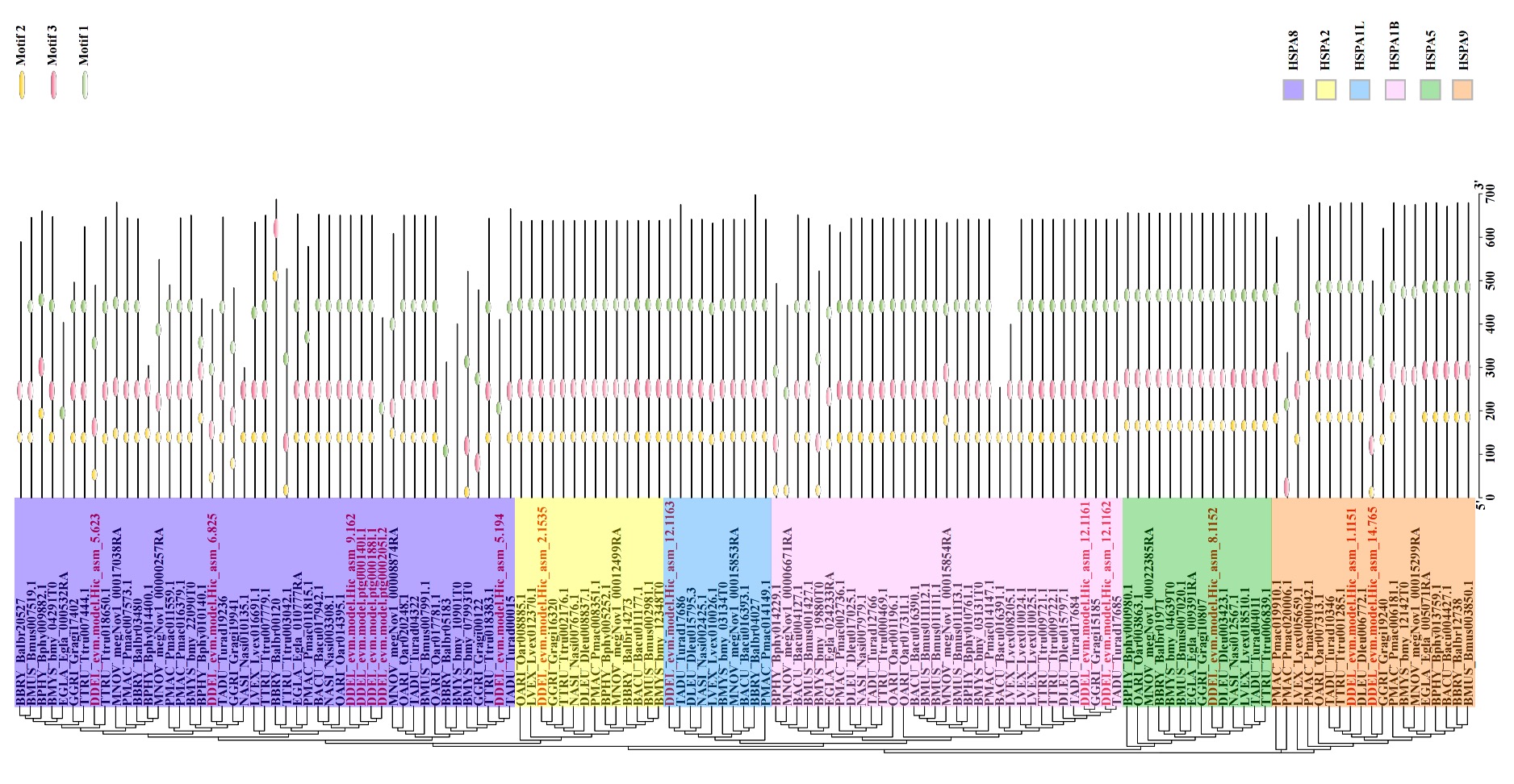
*

**Fig. S6 Phylogenetic analysis of Hsp70 genes across cetacean species using Maximum likelihood, with gene labels derived from the first letter of the genus, the first three letters of the specific name, and the gene number of Hsp70 in the corresponding species.**


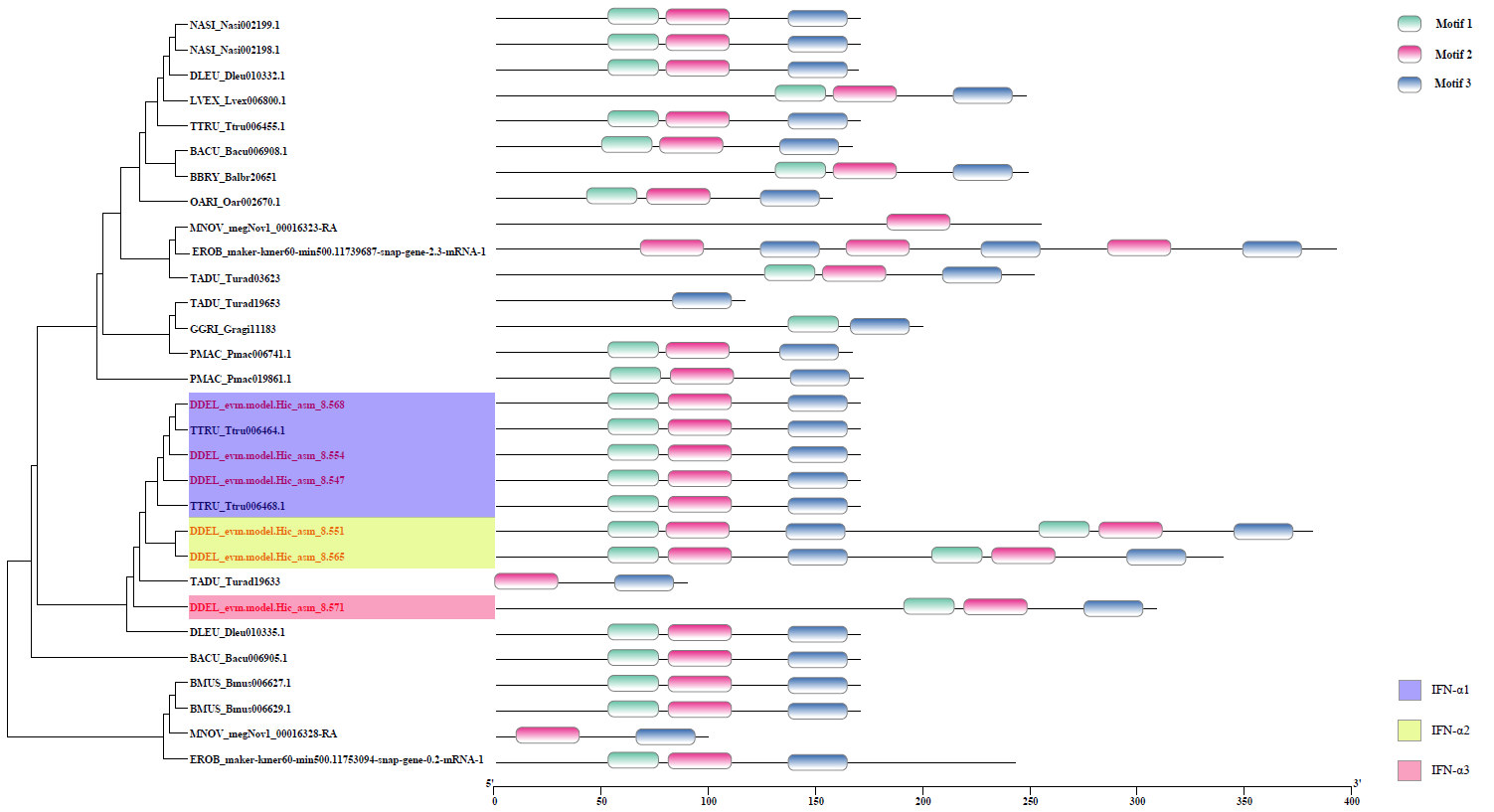


**Fig. S7 Maximum likelihood phylogenetic tree and motifs constructed by IFN-α genes in different species of cetaceans. The gene labels were derived from the first letter of the genus, the first three letters of the specific name, and the gene number of IFN-α in the corresponding species.**


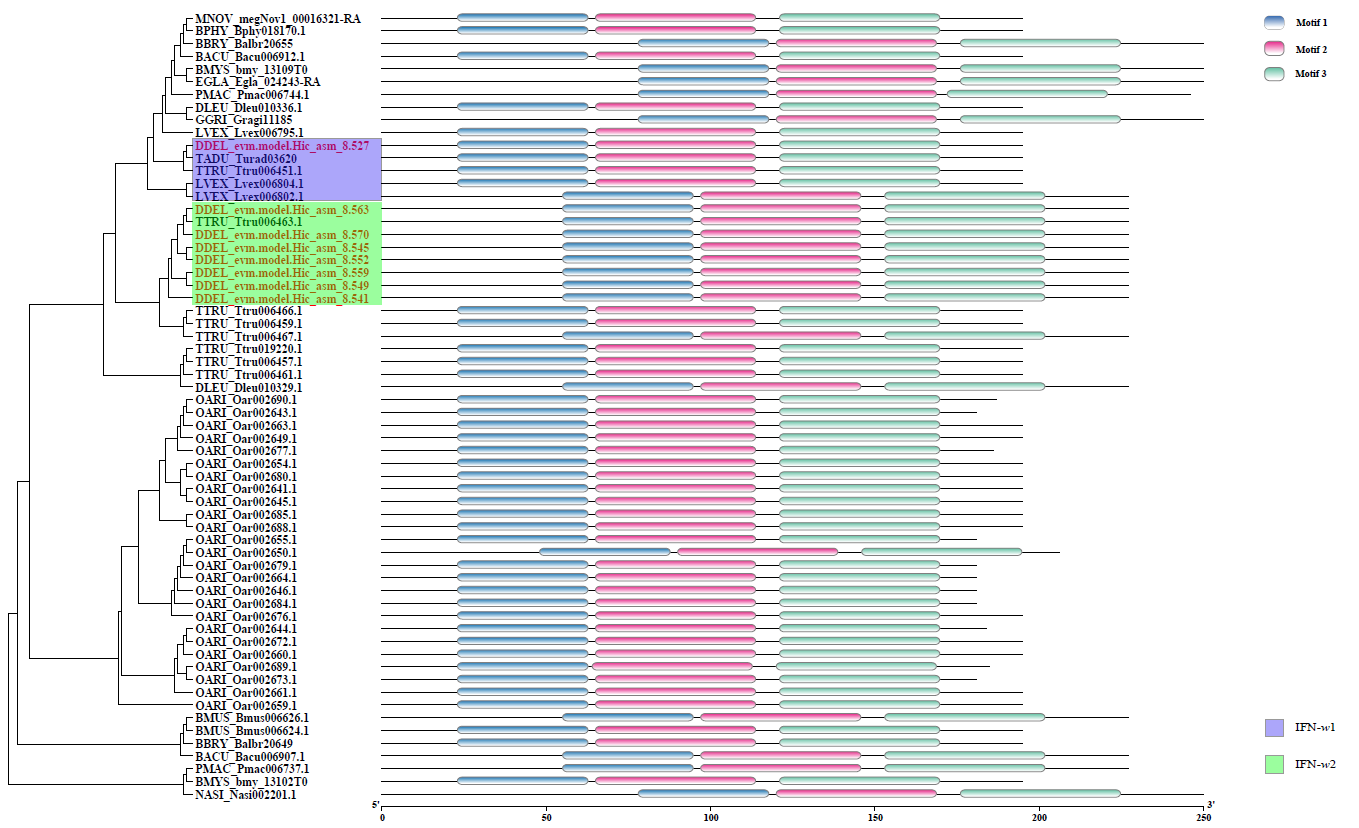


**Fig. S8 Maximum likelihood phylogenetic tree and motifs constructed by IFN-ω genes in different species of cetaceans. The gene labels were derived from the first letter of the genus, the first three letters of the specific name, and the gene number of IFN-ω in the corresponding species.**


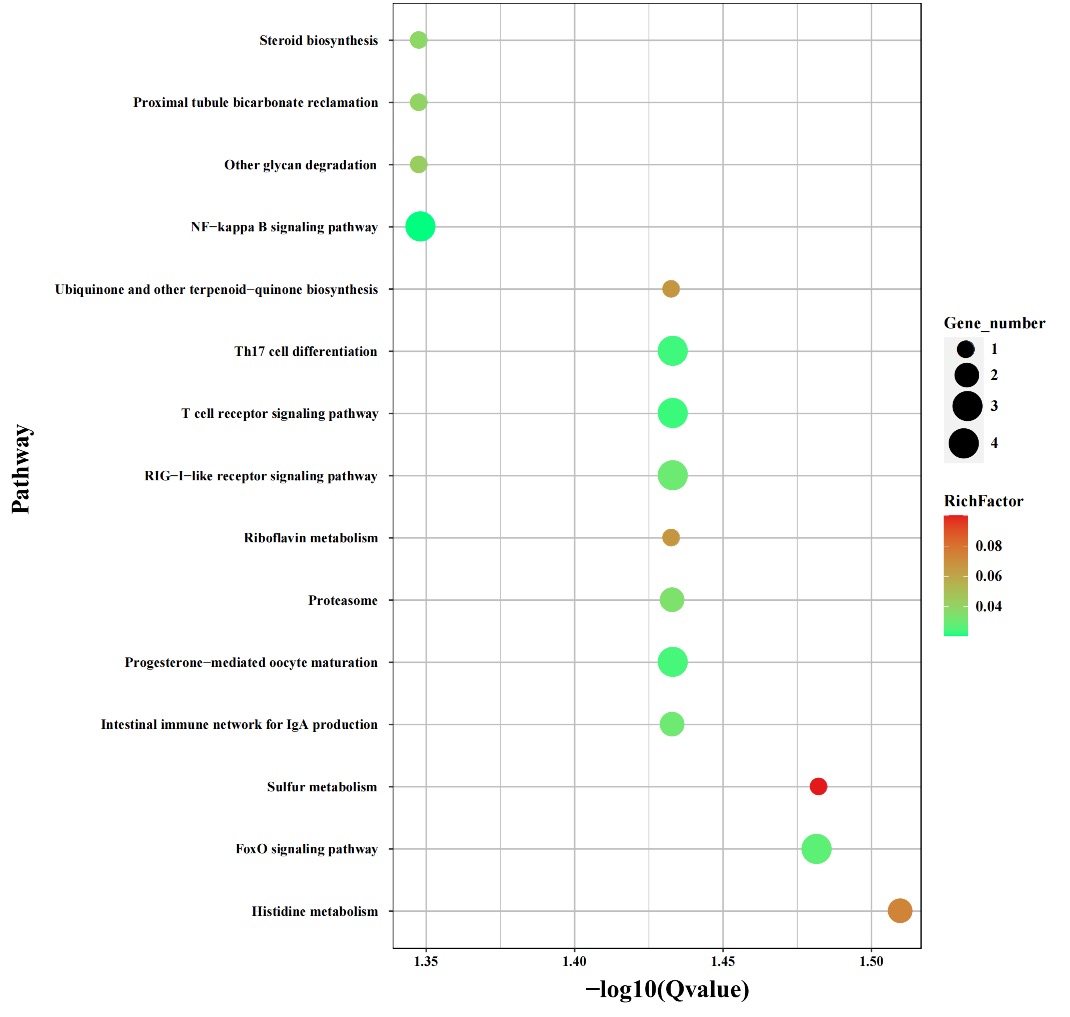


**Fig. S9  KEGG enrichment analysis of significantly positive selection genes in *D. delphis* (*p* < 0.01).**
