# Supplementary material for: Chromosome-level genome provides insights into environmental adaptability and innate immunity in the common dolphin (Delphinus delphis)
Source: BMC Genomics. 2024 Apr 16;25:373. doi: 10.1186/s12864-024-10268-4 (PMC11022445; doi:10.1186/s12864-024-10268-4)
Supplement: Supplementary file 3 — Supplementary Material 3 [file 12864_2024_10268_MOESM3_ESM.docx]

**Table S1 Species information used in annotation and comparative genomics analysis**

| **Species** | **Family** | **Suborder** | **Accession number** | **Source of species images** |
| --- | --- | --- | --- | --- |
| *Physeter macrocephalus* | Physeteridae | Odontoceti | GCF_002837175.2 | <https://cz.wallpapers-fenix.eu/Animals/1/218/> |
| *Tursiops truncatus* | Delphinidae | Odontoceti | GCF_011762595.1 | <https://amazon.clikpic.com/martink/images/20160902-DSC_0549.jpg> |
| *Tursiops aduncus* | Delphinidae | Odontoceti | Yuan et al., 2021 | https://www.monaconatureencyclopedia.com/wp-content/uploads/2014/12/totale._.jpg |
| *Delphinus delphis* | Delphinidae | Odontoceti | Present study | <https://www.dolphin-way.com/dolphins-the-facts/some-dolphin-species/common-dolphin-delphinus-delphis/> |
| *Grampus griseus* | Delphinidae | Odontoceti | Yuan et al., 2021 | https://www.nms.ac.uk/search.axd?command=getcontent&server=Detail&value=PF36959 |
| *Delphinapterus leucas* | Monodontidae | Odontoceti | GCF_002288925.2 | https://www.mediastorehouse.com/p/632/beluga-whale-delphinapterus-leucas-18965133.jpg |
| *Neophocaena asiaeorientalis* | Phocaenidae | Odontoceti | GCF_003031525.2 | https://www.markcarwardine.com/whale_images/MC-FG-00938/thumb1.jpg |
| *Lipotes vexillifer* | Lipotidae | Odontoceti | GCF_000442215.1 | https://www.nms.ac.uk/search.axd?command=getcontent&server=Detail&value=PF32991 |
| *Eubalaena glacialis* | Balaenidae | Mysticeti | https://www.dnazoo.org/assemblies/Eubalaena_glacialis | https://cff2.earth.com/uploads/2017/01/03144058/Eubalaena-glacialis.png |
| *Balaena mysticetus* | Balaenidae | Mysticeti | <http://www.bowhead-whale.org/downloads/> | https://www.mediastorehouse.com/p/632/bowhead-whale-balaena-mysticetus-18965137.jpg |
| *Balaenoptera acutorostrata* | Balaenopteridae | Mysticeti | GCF_000493695.1 | Carwardine, M. (1999). Whales, dolphins, and porpoises (No. Sirsi) i9780816039913). |
| *Balaenoptera brydei* | Balaenopteridae | Mysticeti | Yuan et al., 2021 | http://animal.memozee.com/ArchHAN02/1137161882.jpg |
| *Balaenoptera musculus* | Balaenopteridae | Mysticeti | GCF_009873245.2 | https://www.acsonline.org/assets/images/balaenoptera-musculus.png |
| *Balaenoptera physalus* | Balaenopteridae | Mysticeti | GCA_023338255.1 | http://animal.memozee.com/view.php?tid=5&did=7566&mode=full |
| *Megaptera novaeangliae* | Balaenopteridae | Mysticeti | GCA_004329385.1 | https://img.zcool.cn/community/014425556845c00000012716f4ed1b.jpg@1280w_1l_2o_100sh.jpg |
| *Echrichtius robustus* | Eschrichtiidae | Mysticeti | GCA_002189225.1 | <http://www.mojofun.eu/wp-content/uploads/2017/01/387277_GRAY_WHALE-e1485452433756.jpg> |
| *Ovis aries* | Bovidae | Ruminantia | GCF_016772045.1 | https://www.biolib.cz/cz/taxonimage/id357341/?taxonid=1270257&type=1 |

**Table S2 Correction points used for divergence time estimation**

| **Taxon1** | **Taxon2** | **Divergence time (Mya）** | **CI (max-min) (Mya)** |
| --- | --- | --- | --- |
| *Ovis aries* | Cetacea | 56 | 59-53 |
| *Odontoceti* | Mysticeti | 33.5 | 35.0-31.1 |
| *Delphinidae* | Phocoena | 18.39 | 19.66-16.51 |
| *Balaenopteridae* | Balaenidae | 25.9 | 28.9-22.9 |

**Table S3 Genome statistics of Illumina data removal Duplication**

Sample 1: FDSW200001699-1r_L2; Sample 2: FDSW200001699-1r_L1

| **Sample Name** | **Duplication rate** | **Clean base** | **Non duplication base** | **Clean pairs** | **Non duplication pairs** |
| --- | --- | --- | --- | --- | --- |
| Sample 1 | 16.1548% | 16,458,487,500 | 13,799,647,500 | 54,861,625 | 45,998,825 |
| Sample 2 | 15.4578% | 8,327,550,7500 | 70,402,956,900 | 277,585,025 | 234,676,523 |

**Table S4 Genome characteristics obtained by the Kmer=17 analysis**

| **Kmer** | **Depth** | **Number of kmer** | **Genome size (M)** | **Revised Genome size (M)** | **Heterozygous rate (%)** | **Repeat rate (%)** |
| --- | --- | --- | --- | --- | --- | --- |
| 17 | 29 | 75,216,715,516 | 2,593.68 | 2,569.25 | 0.33 | 57.75 |

**Table S5 Statistical quality of genome data obtained by the platform of Pacbio Sequel II**

| **Read type** | **Read base** | **Read Number** | **Read length (max)** | **Read length (mean)** | **Read length (N50)** |
| --- | --- | --- | --- | --- | --- |
| HiFi reads | 80,797,512,248 | 5,609,073 | 40,800 | 14,404 | 14,840 |

|  |  | **Percentage (%)** |
| --- | --- | --- |
| **Reads** | **Mapping rate** | 99.69 |
| **Genome** | **Average sequencing depth** | 32.60 |
|  | **Coverage** | 99.96 |
|  | **Coverage at least 4×** | 99.83 |
|  | **Coverage at least 10×** | 98.82 |
|  | **Coverage at least 20×** | 90.45 |

**Table S6 Reads coverage statistics of *D. delphis* genome**

**Table S7 SNP statistics of *D. delphis* genome**

|  | **Number** | **Percentage (%)** |
| --- | --- | --- |
| All SNP | 3,038,365 | 0.120176 |
| Heterozygosis SNP | 3,037,537 | 0.120143 |
| Homology SNP | 828 | 3.3e-05 |

**Table S8 CEGMA evaluation of *D. delphis* genome**

| **Complete** | | **Complete + Partial** | |
| --- | --- | --- | --- |
| **Prots** | **Completeness (%)** | **Prots** | **Completeness (%)** |
| 237 | 95.56 | 245 | 98.79 |

Note: ‘Complete’ refers to core genes that are assembled to more than 70% of their full length; ‘Complete + Partial’ encompasses cases where core genes are partially assembled. ‘Prots’ denotes the total number of core eukaryotic genes (CEGs) that have been successfully assembled. ‘Completeness (%)’ indicates the proportion of these assembled core genes relative to the total number of core genes in the CEG database.

**Table S9 BUSCO results of *D. delphis* genome**

| Complete BUSCOs (%) | 91.0 |
| --- | --- |
| Complete and single-copy BUSCOs (%) | 90.0 |
| Complete Duplicated BUSCOs (%) | 1.0 |
| Fragmented BUSCOs (%) | 2.4 |
| Missing BUSCOs (%) | 6.6 |
| Total BUSCO groups searched | 9226 |

**Table S10 Classification of the transposable elements (TEs) in the *D. delphis* genome assembly**

|  | **Denovo+Repbase** | | **TE Proteins** | | **Combined TEs** | |
| --- | --- | --- | --- | --- | --- | --- |
|  | **Length (bp)** | **Percentage in Genome (%)** | **Length (bp)** | **Percentage in Genome (%)** | **Length (bp)** | **Percentage in Genome (%)** |
| DNA | 5,699,196 | 0.22 | 4,002,757 | 0.16 | 8,402,537 | 0.33 |
| LINE | 312,546,830 | 12.21 | 327,558,438 | 12.80 | 485,304,065 | 18.96 |
| SINE | 1,378,191 | 0.05 | 0 | 0 | 1,378,191 | 0.05 |
| LTR | 784,358,537 | 30.65 | 4,305,925 | 0.17 | 786,101,305 | 30.72 |
| Unknown | 21,038,202 | 0.82 | 0 | 0 | 21,038,202 | 0.82 |
| Total | 1,051,100,870 | 41.07 | 335,852,965 | 13.12 | 1,058,725,214 | 41.37 |

**Table S11 The statistical result of non-coding RNA in *D. delphis* genome**

| Type | | Copy number | Average length (bp) | Total length (bp) | Percentage of genome (%) |
| --- | --- | --- | --- | --- | --- |
| miRNA | | 24,996 | 97.76 | 2,443,617 | 0.095488 |
| tRNA | | 16,963 | 72.90 | 1,236,519 | 0.048319 |
| rRNA | rRNA | 252 | 162.87 | 41,044 | 0.001604 |
|  | 18S | 190 | 183.16 | 34,800 | 0.001360 |
|  | 28S | 62 | 100.71 | 6,244 | 0.000244 |
|  | 5.8S | 0 | 0 | 0 | 0 |
|  | 5S | 0 | 0 | 0 | 0 |
| snRNA | snRNA | 1,874 | 114.84 | 215,214 | 0.008410 |
|  | CD-box | 378 | 93.13 | 35,202 | 0.001376 |
|  | HACA-box | 333 | 134.94 | 44,934 | 0.001756 |
|  | splicing | 1,098 | 113.87 | 125,032 | 0.004886 |
|  | scaRNA | 56 | 169.50 | 9,492 | 0.000371 |
|  | Unknown | 9 | 61.56 | 554 | 0.000022 |

**Table S12 Characteristics of chromosomes of *D. delphis.***

| **Chr** | **Gene Number** | **Chr Length (bp)** | **Gene Density (genes/Mb)** |
| --- | --- | --- | --- |
| 1 | 2,149 | 183,537,782 | 11.71 |
| 2 | 1,558 | 181,113,018 | 8.60 |
| 3 | 1,596 | 174,782,813 | 9.13 |
| 4 | 993 | 144,765,131 | 6.86 |
| 5 | 888 | 138,636,026 | 6.41 |
| 6 | 1,140 | 116,210,877 | 9.81 |
| 7 | 743 | 123,474,987 | 6.02 |
| 8 | 1,247 | 108,933,991 | 11.45 |
| 9 | 803 | 105,551,416 | 7.61 |
| 10 | 1,141 | 105,416,818 | 10.82 |
| 11 | 1,108 | 105,186,520 | 10.53 |
| 12 | 717 | 90,270,747 | 7.94 |
| 13 | 849 | 90,124,573 | 9.42 |
| 14 | 527 | 93,306,678 | 5.65 |
| 15 | 1,333 | 90,076,404 | 14.80 |
| 16 | 720 | 86,192,151 | 8.35 |
| 17 | 550 | 82,189,745 | 6.69 |
| 18 | 432 | 79,809,380 | 5.41 |
| 19 | 1,212 | 59,779,654 | 20.27 |
| 20 | 1,125 | 60,795,635 | 18.50 |
| 21 | 232 | 40,267,883 | 5.76 |
| X | 984 | 124,379,003 | 7.91 |
| Y | 90 | 15,991,517 | 5.63 |
| Total | 22,137 | 2,400,792,749 | 9.22 |

**Table S13 Statistics of gene family clustering results of all the species.**

| **Species** | **Genes number** | **Unclustered genes** | **Genes in families** | **Family number** | **Unique families** | **Unique families genes** | **Common families** | **Common families genes** | **Single copy** | **Single copy genes** | **Average genes per family** |
| --- | --- | --- | --- | --- | --- | --- | --- | --- | --- | --- | --- |
| *D. delphis* | 22148 | 4115 | 18033 | 14533 | 32 | 69 | 5939 | 8591 | 2630 | 2630 | 1.241 |
| *T. truncatus* | 19227 | 333 | 18894 | 15019 | 6 | 17 | 5939 | 8853 | 2630 | 2630 | 1.258 |
| *T. aduncus* | 20188 | 803 | 19385 | 15592 | 10 | 24 | 5939 | 8743 | 2630 | 2630 | 1.243 |
| *G. griseus* | 20534 | 767 | 19767 | 14790 | 41 | 90 | 5939 | 9362 | 2630 | 2630 | 1.337 |
| *D. leucas* | 18456 | 231 | 18225 | 14800 | 0 | 0 | 5939 | 8723 | 2630 | 2630 | 1.231 |
| *B. acutorostrata* | 18819 | 269 | 18550 | 14884 | 5 | 11 | 5939 | 8793 | 2630 | 2630 | 1.246 |
| *B. musculus* | 19673 | 465 | 19208 | 15178 | 2 | 4 | 5939 | 8969 | 2630 | 2630 | 1.266 |
| *B. physalus* | 19655 | 1777 | 17878 | 14618 | 3 | 6 | 5939 | 8546 | 2630 | 2630 | 1.223 |
| *B. brydei* | 20809 | 1106 | 19703 | 14351 | 40 | 1449 | 5939 | 8409 | 2630 | 2630 | 1.373 |
| *M. novaeangliae* | 22980 | 2434 | 20546 | 16441 | 8 | 17 | 5939 | 9048 | 2630 | 2630 | 1.25 |
| *B. mysticetus* | 22733 | 2733 | 20000 | 16099 | 3 | 6 | 5939 | 8944 | 2630 | 2630 | 1.242 |
| *E. glacialis* | 25132 | 5365 | 19767 | 15614 | 118 | 308 | 5939 | 8856 | 2630 | 2630 | 1.266 |
| *E. robustus* | 22711 | 6642 | 16069 | 12433 | 13 | 26 | 5939 | 8846 | 2630 | 2630 | 1.292 |
| *L. vexillifer* | 18877 | 655 | 18222 | 14539 | 157 | 432 | 5939 | 8667 | 2630 | 2630 | 1.253 |
| *N. asiaeorientalis* | 18245 | 270 | 17975 | 14633 | 1 | 2 | 5939 | 8666 | 2630 | 2630 | 1.228 |
| *P. macrocephalus* | 20161 | 1151 | 19010 | 15004 | 78 | 172 | 5939 | 9039 | 2630 | 2630 | 1.267 |
| *O. aries* | 21244 | 1150 | 20094 | 14875 | 126 | 659 | 5939 | 9051 | 2630 | 2630 | 1.351 |

**Table S14 Comparative sequence identity analysis for HSP70 gene members.**

| **Query Sequence Name** | **Subject Sequence Name** | **Identity (%)** | **Alignment Length (bp)** | **E-value** |
| --- | --- | --- | --- | --- |
| evm.model.Hic_asm_9.162 | evm.model.ptg000140l.1 | 99.693 | 651 | 0 |
| evm.model.Hic_asm_9.162 | evm.model.ptg000188l.1 | 99.232 | 651 | 0 |
| evm.model.Hic_asm_9.162 | evm.model.Hic_asm_12.1162 | 83.871 | 651 | 0 |
| evm.model.Hic_asm_9.162 | evm.model.Hic_asm_2.1535 | 87.276 | 613 | 0 |
| evm.model.Hic_asm_9.162 | evm.model.Hic_asm_12.1161 | 83.717 | 651 | 0 |
| evm.model.Hic_asm_9.162 | evm.model.Hic_asm_12.1163 | 81.231 | 650 | 0 |
| evm.model.Hic_asm_9.162 | evm.model.Hic_asm_5.623 | 91.837 | 490 | 0 |
| evm.model.Hic_asm_9.162 | evm.model.ptg000205l.2 | 99.277 | 415 | 0 |
| evm.model.Hic_asm_9.162 | evm.model.Hic_asm_6.825 | 82.957 | 487 | 0 |
| evm.model.Hic_asm_9.162 | evm.model.Hic_asm_5.194 | 92.289 | 415 | 0 |
| evm.model.Hic_asm_5.194 | evm.model.Hic_asm_9.162 | 93.369 | 377 | 0 |
| evm.model.Hic_asm_5.194 | evm.model.ptg000140l.1 | 93.103 | 377 | 0 |
| evm.model.Hic_asm_5.194 | evm.model.ptg000205l.2 | 92.838 | 377 | 0 |
| evm.model.Hic_asm_5.194 | evm.model.ptg000188l.1 | 92.838 | 377 | 0 |
| evm.model.Hic_asm_5.194 | evm.model.Hic_asm_2.1535 | 80.585 | 376 | 0 |
| evm.model.Hic_asm_5.194 | evm.model.Hic_asm_5.623 | 84.956 | 339 | 0 |
| evm.model.Hic_asm_5.623 | evm.model.ptg000188l.1 | 91.837 | 490 | 0 |
| evm.model.Hic_asm_5.623 | evm.model.Hic_asm_9.162 | 91.837 | 490 | 0 |
| evm.model.Hic_asm_5.623 | evm.model.ptg000140l.1 | 91.633 | 490 | 0 |
| evm.model.Hic_asm_5.623 | evm.model.ptg000205l.2 | 90.56 | 339 | 0 |
| evm.model.Hic_asm_5.623 | evm.model.Hic_asm_5.194 | 84.956 | 339 | 0 |
| evm.model.Hic_asm_2.1535 | evm.model.Hic_asm_9.162 | 87.276 | 613 | 0 |
| evm.model.Hic_asm_2.1535 | evm.model.ptg000140l.1 | 87.113 | 613 | 0 |
| evm.model.Hic_asm_2.1535 | evm.model.ptg000188l.1 | 86.623 | 613 | 0 |
| evm.model.Hic_asm_2.1535 | evm.model.Hic_asm_12.1162 | 82.253 | 648 | 0 |
| evm.model.Hic_asm_2.1535 | evm.model.Hic_asm_12.1161 | 82.253 | 648 | 0 |
| evm.model.Hic_asm_2.1535 | evm.model.Hic_asm_12.1163 | 80.062 | 642 | 0 |
| evm.model.Hic_asm_2.1535 | evm.model.ptg000205l.2 | 85.676 | 377 | 0 |
| evm.model.ptg000140l.1 | evm.model.Hic_asm_9.162 | 99.693 | 651 | 0 |
| evm.model.ptg000140l.1 | evm.model.ptg000188l.1 | 99.539 | 651 | 0 |
| evm.model.ptg000140l.1 | evm.model.Hic_asm_12.1162 | 83.717 | 651 | 0 |
| evm.model.ptg000140l.1 | evm.model.Hic_asm_2.1535 | 87.113 | 613 | 0 |
| evm.model.ptg000140l.1 | evm.model.Hic_asm_12.1161 | 83.564 | 651 | 0 |
| evm.model.ptg000140l.1 | evm.model.Hic_asm_12.1163 | 81.077 | 650 | 0 |
| evm.model.ptg000140l.1 | evm.model.Hic_asm_5.623 | 91.633 | 490 | 0 |
| evm.model.ptg000140l.1 | evm.model.ptg000205l.2 | 99.759 | 415 | 0 |
| evm.model.ptg000140l.1 | evm.model.Hic_asm_6.825 | 82.752 | 487 | 0 |
| evm.model.ptg000140l.1 | evm.model.Hic_asm_5.194 | 92.048 | 415 | 0 |
| evm.model.Hic_asm_6.825 | evm.model.Hic_asm_9.162 | 82.957 | 487 | 0 |
| evm.model.Hic_asm_6.825 | evm.model.ptg000188l.1 | 82.752 | 487 | 0 |
| evm.model.Hic_asm_6.825 | evm.model.ptg000140l.1 | 82.752 | 487 | 0 |
| evm.model.Hic_asm_14.765 | evm.model.Hic_asm_1.1151 | 96.115 | 489 | 0 |
| evm.model.Hic_asm_1.1151 | evm.model.Hic_asm_14.765 | 96.115 | 489 | 0 |
| evm.model.Hic_asm_12.1161 | evm.model.Hic_asm_12.1162 | 99.688 | 642 | 0 |
| evm.model.Hic_asm_12.1161 | evm.model.Hic_asm_12.1163 | 88.612 | 641 | 0 |
| evm.model.Hic_asm_12.1161 | evm.model.Hic_asm_9.162 | 86.275 | 612 | 0 |
| evm.model.Hic_asm_12.1161 | evm.model.ptg000140l.1 | 86.111 | 612 | 0 |
| evm.model.Hic_asm_12.1161 | evm.model.ptg000188l.1 | 85.621 | 612 | 0 |
| evm.model.Hic_asm_12.1161 | evm.model.Hic_asm_2.1535 | 82.426 | 643 | 0 |
| evm.model.Hic_asm_12.1161 | evm.model.ptg000205l.2 | 84.615 | 377 | 0 |
| evm.model.Hic_asm_12.1162 | evm.model.Hic_asm_12.1161 | 99.688 | 642 | 0 |
| evm.model.Hic_asm_12.1162 | evm.model.Hic_asm_12.1163 | 88.612 | 641 | 0 |
| evm.model.Hic_asm_12.1162 | evm.model.Hic_asm_9.162 | 86.275 | 612 | 0 |
| evm.model.Hic_asm_12.1162 | evm.model.ptg000140l.1 | 86.111 | 612 | 0 |
| evm.model.Hic_asm_12.1162 | evm.model.ptg000188l.1 | 85.621 | 612 | 0 |
| evm.model.Hic_asm_12.1162 | evm.model.Hic_asm_2.1535 | 82.582 | 643 | 0 |
| evm.model.Hic_asm_12.1162 | evm.model.ptg000205l.2 | 84.615 | 377 | 0 |
| evm.model.Hic_asm_12.1163 | evm.model.Hic_asm_12.1162 | 88.144 | 641 | 0 |
| evm.model.Hic_asm_12.1163 | evm.model.Hic_asm_12.1161 | 88.144 | 641 | 0 |
| evm.model.Hic_asm_12.1163 | evm.model.Hic_asm_9.162 | 83.797 | 611 | 0 |
| evm.model.Hic_asm_12.1163 | evm.model.ptg000140l.1 | 83.633 | 611 | 0 |
| evm.model.Hic_asm_12.1163 | evm.model.ptg000188l.1 | 83.142 | 611 | 0 |
| evm.model.Hic_asm_12.1163 | evm.model.Hic_asm_2.1535 | 80.062 | 642 | 0 |
| evm.model.Hic_asm_12.1163 | evm.model.ptg000205l.2 | 81.167 | 377 | 0 |
| evm.model.ptg000205l.2 | evm.model.ptg000140l.1 | 99.759 | 415 | 0 |
| evm.model.ptg000205l.2 | evm.model.Hic_asm_9.162 | 99.277 | 415 | 0 |
| evm.model.ptg000205l.2 | evm.model.ptg000188l.1 | 99.518 | 415 | 0 |
| evm.model.ptg000205l.2 | evm.model.Hic_asm_5.194 | 91.807 | 415 | 0 |
| evm.model.ptg000205l.2 | evm.model.Hic_asm_12.1162 | 81.205 | 415 | 0 |
| evm.model.ptg000205l.2 | evm.model.Hic_asm_12.1161 | 80.964 | 415 | 0 |
| evm.model.ptg000205l.2 | evm.model.Hic_asm_2.1535 | 85.904 | 376 | 0 |
| evm.model.ptg000205l.2 | evm.model.Hic_asm_5.623 | 90.56 | 339 | 0 |
| evm.model.ptg000188l.1 | evm.model.ptg000140l.1 | 99.539 | 651 | 0 |
| evm.model.ptg000188l.1 | evm.model.Hic_asm_9.162 | 99.232 | 651 | 0 |
| evm.model.ptg000188l.1 | evm.model.Hic_asm_2.1535 | 86.623 | 613 | 0 |
| evm.model.ptg000188l.1 | evm.model.Hic_asm_12.1162 | 83.257 | 651 | 0 |
| evm.model.ptg000188l.1 | evm.model.Hic_asm_12.1161 | 83.103 | 651 | 0 |
| evm.model.ptg000188l.1 | evm.model.Hic_asm_12.1163 | 80.615 | 650 | 0 |
| evm.model.ptg000188l.1 | evm.model.Hic_asm_5.623 | 91.837 | 490 | 0 |
| evm.model.ptg000188l.1 | evm.model.ptg000205l.2 | 99.518 | 415 | 0 |
| evm.model.ptg000188l.1 | evm.model.Hic_asm_6.825 | 82.752 | 487 | 0 |
| evm.model.ptg000188l.1 | evm.model.Hic_asm_5.194 | 91.807 | 415 | 0 |

**Table S15 Comparative sequence identity analysis for IFN-α gene members**

| **Query Sequence Name** | **Subject Sequence Name** | **Identity (%)** | **Alignment Length (bp)** | **E-value** |
| --- | --- | --- | --- | --- |
| evm.model.Hic_asm_8.547 | evm.model.Hic_asm_8.568 | 100 | 171 | 2.64E-132 |
| evm.model.Hic_asm_8.547 | evm.model.Hic_asm_8.554 | 100 | 171 | 2.64E-132 |
| evm.model.Hic_asm_8.547 | evm.model.Hic_asm_8.571 | 98.225 | 169 | 7.12E-129 |
| evm.model.Hic_asm_8.547 | evm.model.Hic_asm_8.565 | 98.81 | 168 | 1.15E-127 |
| evm.model.Hic_asm_8.547 | evm.model.Hic_asm_8.551 | 98.824 | 170 | 1.39E-127 |
| evm.model.Hic_asm_8.554 | evm.model.Hic_asm_8.568 | 100 | 171 | 2.64E-132 |
| evm.model.Hic_asm_8.554 | evm.model.Hic_asm_8.547 | 100 | 171 | 2.64E-132 |
| evm.model.Hic_asm_8.554 | evm.model.Hic_asm_8.571 | 98.225 | 169 | 7.12E-129 |
| evm.model.Hic_asm_8.554 | evm.model.Hic_asm_8.565 | 98.81 | 168 | 1.15E-127 |
| evm.model.Hic_asm_8.554 | evm.model.Hic_asm_8.551 | 98.824 | 170 | 1.39E-127 |
| evm.model.Hic_asm_8.551 | evm.model.Hic_asm_8.565 | 86.126 | 382 | 0 |
| evm.model.Hic_asm_8.551 | evm.model.Hic_asm_8.568 | 98.824 | 170 | 3.09E-127 |
| evm.model.Hic_asm_8.551 | evm.model.Hic_asm_8.554 | 98.824 | 170 | 3.09E-127 |
| evm.model.Hic_asm_8.551 | evm.model.Hic_asm_8.547 | 98.824 | 170 | 3.09E-127 |
| evm.model.Hic_asm_8.551 | evm.model.Hic_asm_8.571 | 97.024 | 168 | 3.19E-123 |
| evm.model.Hic_asm_8.568 | evm.model.Hic_asm_8.554 | 100 | 171 | 2.64E-132 |
| evm.model.Hic_asm_8.568 | evm.model.Hic_asm_8.547 | 100 | 171 | 2.64E-132 |
| evm.model.Hic_asm_8.568 | evm.model.Hic_asm_8.571 | 98.225 | 169 | 7.12E-129 |
| evm.model.Hic_asm_8.568 | evm.model.Hic_asm_8.565 | 98.81 | 168 | 1.15E-127 |
| evm.model.Hic_asm_8.568 | evm.model.Hic_asm_8.551 | 98.824 | 170 | 1.39E-127 |
| evm.model.Hic_asm_8.571 | evm.model.Hic_asm_8.568 | 98.225 | 169 | 1.29E-128 |
| evm.model.Hic_asm_8.571 | evm.model.Hic_asm_8.554 | 98.225 | 169 | 1.29E-128 |
| evm.model.Hic_asm_8.571 | evm.model.Hic_asm_8.547 | 98.225 | 169 | 1.29E-128 |
| evm.model.Hic_asm_8.571 | evm.model.Hic_asm_8.565 | 96.988 | 166 | 6.33E-124 |
| evm.model.Hic_asm_8.571 | evm.model.Hic_asm_8.551 | 97.024 | 168 | 2.58E-123 |
| evm.model.Hic_asm_8.565 | evm.model.Hic_asm_8.551 | 86.387 | 382 | 0 |
| evm.model.Hic_asm_8.565 | evm.model.Hic_asm_8.568 | 98.81 | 168 | 2.29E-127 |
| evm.model.Hic_asm_8.565 | evm.model.Hic_asm_8.554 | 98.81 | 168 | 2.29E-127 |
| evm.model.Hic_asm_8.565 | evm.model.Hic_asm_8.547 | 98.81 | 168 | 2.29E-127 |
| evm.model.Hic_asm_8.565 | evm.model.Hic_asm_8.571 | 96.988 | 166 | 6.97E-124 |

**Table S16 Comparative sequence identity analysis for IFN-ω gene members**

| **Query Sequence Name** | **Subject Sequence Name** | **Identity (%)** | **Alignment Length (bp)** | **E-value** |
| --- | --- | --- | --- | --- |
| evm.model.Hic_asm_8.563 | evm.model.Hic_asm_8.545 | 99.561 | 228 | 3.26E-174 |
| evm.model.Hic_asm_8.563 | evm.model.Hic_asm_8.552 | 99.561 | 228 | 3.26E-174 |
| evm.model.Hic_asm_8.563 | evm.model.Hic_asm_8.570 | 99.561 | 228 | 5.96E-174 |
| evm.model.Hic_asm_8.563 | evm.model.Hic_asm_8.559 | 99.123 | 228 | 9.99E-174 |
| evm.model.Hic_asm_8.563 | evm.model.Hic_asm_8.549 | 99.123 | 228 | 9.99E-174 |
| evm.model.Hic_asm_8.563 | evm.model.Hic_asm_8.541 | 98.684 | 228 | 2.99E-173 |
| evm.model.Hic_asm_8.563 | evm.model.Hic_asm_8.527 | 95.408 | 196 | 8.14E-142 |
| evm.model.Hic_asm_8.541 | evm.model.Hic_asm_8.559 | 99.561 | 228 | 5.63E-175 |
| evm.model.Hic_asm_8.541 | evm.model.Hic_asm_8.549 | 99.561 | 228 | 5.63E-175 |
| evm.model.Hic_asm_8.541 | evm.model.Hic_asm_8.545 | 99.123 | 228 | 2.32E-174 |
| evm.model.Hic_asm_8.541 | evm.model.Hic_asm_8.552 | 99.123 | 228 | 2.32E-174 |
| evm.model.Hic_asm_8.541 | evm.model.Hic_asm_8.563 | 98.684 | 228 | 2.99E-173 |
| evm.model.Hic_asm_8.541 | evm.model.Hic_asm_8.570 | 98.246 | 228 | 3.31E-172 |
| evm.model.Hic_asm_8.541 | evm.model.Hic_asm_8.527 | 95.918 | 196 | 1.89E-142 |
| evm.model.Hic_asm_8.549 | evm.model.Hic_asm_8.559 | 100 | 228 | 1.51E-175 |
| evm.model.Hic_asm_8.549 | evm.model.Hic_asm_8.541 | 99.561 | 228 | 5.63E-175 |
| evm.model.Hic_asm_8.549 | evm.model.Hic_asm_8.545 | 99.561 | 228 | 7.92E-175 |
| evm.model.Hic_asm_8.549 | evm.model.Hic_asm_8.552 | 99.561 | 228 | 7.92E-175 |
| evm.model.Hic_asm_8.549 | evm.model.Hic_asm_8.563 | 99.123 | 228 | 9.99E-174 |
| evm.model.Hic_asm_8.549 | evm.model.Hic_asm_8.570 | 98.684 | 228 | 1.05E-172 |
| evm.model.Hic_asm_8.549 | evm.model.Hic_asm_8.527 | 95.408 | 196 | 1.02E-141 |
| evm.model.Hic_asm_8.552 | evm.model.Hic_asm_8.545 | 100 | 228 | 2.59E-175 |
| evm.model.Hic_asm_8.552 | evm.model.Hic_asm_8.559 | 99.561 | 228 | 7.92E-175 |
| evm.model.Hic_asm_8.552 | evm.model.Hic_asm_8.549 | 99.561 | 228 | 7.92E-175 |
| evm.model.Hic_asm_8.552 | evm.model.Hic_asm_8.541 | 99.123 | 228 | 2.32E-174 |
| evm.model.Hic_asm_8.552 | evm.model.Hic_asm_8.563 | 99.561 | 228 | 3.26E-174 |
| evm.model.Hic_asm_8.552 | evm.model.Hic_asm_8.570 | 99.123 | 228 | 3.49E-173 |
| evm.model.Hic_asm_8.552 | evm.model.Hic_asm_8.527 | 94.898 | 196 | 5.43E-141 |
| evm.model.Hic_asm_8.559 | evm.model.Hic_asm_8.549 | 100 | 228 | 1.51E-175 |
| evm.model.Hic_asm_8.559 | evm.model.Hic_asm_8.541 | 99.561 | 228 | 5.63E-175 |
| evm.model.Hic_asm_8.559 | evm.model.Hic_asm_8.545 | 99.561 | 228 | 7.92E-175 |
| evm.model.Hic_asm_8.559 | evm.model.Hic_asm_8.552 | 99.561 | 228 | 7.92E-175 |
| evm.model.Hic_asm_8.559 | evm.model.Hic_asm_8.563 | 99.123 | 228 | 9.99E-174 |
| evm.model.Hic_asm_8.559 | evm.model.Hic_asm_8.570 | 98.684 | 228 | 1.05E-172 |
| evm.model.Hic_asm_8.559 | evm.model.Hic_asm_8.527 | 95.408 | 196 | 1.02E-141 |
| evm.model.Hic_asm_8.527 | evm.model.Hic_asm_8.570 | 95.918 | 196 | 1.13E-142 |
| evm.model.Hic_asm_8.527 | evm.model.Hic_asm_8.541 | 95.918 | 196 | 1.63E-142 |
| evm.model.Hic_asm_8.527 | evm.model.Hic_asm_8.563 | 95.408 | 196 | 7.00E-142 |
| evm.model.Hic_asm_8.527 | evm.model.Hic_asm_8.559 | 95.408 | 196 | 8.81E-142 |
| evm.model.Hic_asm_8.527 | evm.model.Hic_asm_8.549 | 95.408 | 196 | 8.81E-142 |
| evm.model.Hic_asm_8.527 | evm.model.Hic_asm_8.545 | 94.898 | 196 | 4.67E-141 |
| evm.model.Hic_asm_8.527 | evm.model.Hic_asm_8.552 | 94.898 | 196 | 4.67E-141 |
| evm.model.Hic_asm_8.545 | evm.model.Hic_asm_8.552 | 100 | 228 | 2.59E-175 |
| evm.model.Hic_asm_8.545 | evm.model.Hic_asm_8.559 | 99.561 | 228 | 7.92E-175 |
| evm.model.Hic_asm_8.545 | evm.model.Hic_asm_8.549 | 99.561 | 228 | 7.92E-175 |
| evm.model.Hic_asm_8.545 | evm.model.Hic_asm_8.541 | 99.123 | 228 | 2.32E-174 |
| evm.model.Hic_asm_8.545 | evm.model.Hic_asm_8.563 | 99.561 | 228 | 3.26E-174 |
| evm.model.Hic_asm_8.545 | evm.model.Hic_asm_8.570 | 99.123 | 228 | 3.49E-173 |
| evm.model.Hic_asm_8.545 | evm.model.Hic_asm_8.527 | 94.898 | 196 | 5.43E-141 |
| evm.model.Hic_asm_8.570 | evm.model.Hic_asm_8.563 | 99.561 | 228 | 5.96E-174 |
| evm.model.Hic_asm_8.570 | evm.model.Hic_asm_8.545 | 99.123 | 228 | 3.49E-173 |
| evm.model.Hic_asm_8.570 | evm.model.Hic_asm_8.552 | 99.123 | 228 | 3.49E-173 |
| evm.model.Hic_asm_8.570 | evm.model.Hic_asm_8.559 | 98.684 | 228 | 1.05E-172 |
| evm.model.Hic_asm_8.570 | evm.model.Hic_asm_8.549 | 98.684 | 228 | 1.05E-172 |
| evm.model.Hic_asm_8.570 | evm.model.Hic_asm_8.541 | 98.246 | 228 | 3.31E-172 |
| evm.model.Hic_asm_8.570 | evm.model.Hic_asm_8.527 | 95.918 | 196 | 1.32E-142 |
